# Optimisation of Surface-Initiated Photoiniferter-Mediated Polymerisation under Confinement, and the Formation of Block Copolymers in Mesoporous Films

**DOI:** 10.3390/polym9100539

**Published:** 2017-10-23

**Authors:** Jessica C. Tom, Robert Brilmayer, Johannes Schmidt, Annette Andrieu-Brunsen

**Affiliations:** 1 Ernst-Berl Institut für Technische und Makromolekulare Chemie, Technische Universität Darmstadt, Alarich-Weiss-Straße 4, 64287 Darmstadt, Germany; jessica.c.h.tom@gmail.com (J.C.T.); brilmayer@cellulose.tu-darmstadt.de (R.B.); 2 Technische Universität Berlin, Fakultät II, Institut für Chemie, Hardenbergstr. 40, 10623 Berlin, Germany; johannes.schmidt@tu-berlin.de

**Keywords:** grafting from, surface-initiated, photoiniferter, photoinitiated, block copolymers, mesoporous silica, ionic permselectivity, local functionalisation

## Abstract

Nature as the ultimate inspiration can direct, gate, and selectively transport species across channels to fulfil a specific targeted function. Harnessing such precision over local structure and functionality at the nanoscale is expected to lead to indispensable developments in synthetic channels for application in catalysis, filtration and sensing, and in drug delivery. By combining mesoporous materials with localised charge-switchable poly(2-(dimethylamino)ethyl methacrylate) (PDMAEMA) brushes, precisely controlling pore filling and exploring the possibility of incorporating two different responsive polymers, we hope to approach the precision control of natural systems in the absence of an external force. Here, we report a simple one-step approach to prepare a mesoporous silica thin film with ~8 nm pores functionalised with a photoiniferter by combining sol–gel chemistry and evaporation-induced self-assembly (EISA). We show that surface-initiated photoiniferter-mediated polymerisation (SI-PIMP) allows the incorporation of a high polymer content up to geometrical pore blocking by the simple application of UV light in the presence of a monomer and solvent, proceeding in a controlled manner in pore sizes below 10 nm, with the potential to tune the material properties through the formation of surface-grafted block copolymers.

## 1. Introduction

The high degree of control exercised in biological channels to regulate key processes including ionic flow and molecular transport across cell membranes is the ultimate inspiration and key motivator for the material scientist to mimic in synthetic materials [1,2]. To achieve this, precise control over the structure, functional density, and ideally local placement is required [2].

Ordered mesoporous silicas (OMSs) are an ideal support structure due to their high specific surface area, dimensional stability, and their uniform, ordered channel structure with tuneable pore size and geometry [3,4]. Furthermore, the rich surface hydroxyl groups available on the silica surface are a convenient handle for various synthetic transformations [3]. These properties have afforded OMSs significant research interest over the last several decades, attracting attention for application in a broad range of fields from catalysis to controlled drug delivery [2,4,5,6,7,8].

The incorporation of polymeric architectures offers the greatest amount of versatility and a high degree of functionality; e.g., a high density of chargeable groups can be incorporated into the porous structure [9]. The incorporation of a polymer coating further permits the intelligent design of hybrid materials. For example, the careful selection of monomers can endow the hybrid material with sensitivity towards a number of external stimuli including pH, redox potential, temperature, solvent, salt concentration, and even light to create “smart” materials, changing their transport behaviour by altering the ion–pore wall interactions [2,9].

Such responsive materials possess great potential for various applications [5]: catalysis and nanoreactors [10], energy storage [11], molecular separation and filtration [12,13,14], controlled drug delivery and release [15], and in microfluidic devices from chemical sensing and biosensing [2] to lab-on-chip devices (pre-concentration, enrichment/extraction for analysis, e.g., pesticides) [16].

Polymerisation in spatially confined mesoporous materials is a key technology that has been widely investigated over the last decade with advances in both soft chemistry and controlled/“living” radical polymerisation (CLRP) techniques [7,8,17,18]. A number of CLRP techniques have been successfully applied to the functionalisation of mesoporous silica including atom transfer radical polymerisation (ATRP) [19,20,21], reversible addition‒fragmentation chain transfer (RAFT) polymerisation [22], nitroxide-mediated radical polymerisation (NMP) [23], and photoiniferter-mediated polymerisation (PIMP) [23,24] through a grafting from or surface-initiated approach. While the successful grafting of pre-formed polymer to the interior and exterior surface has been reported in large-pore (15 nm) membranes [25], this approach is preferred for gated membranes with sterics limiting the molecular weight, grafting density, and pore filling achievable [8,17,22,26].

The formation of polymer brushes from the interior surface of mesopores through surface-initiated polymerisation, while synthetically more viable than post-grafting pre-formed polymers to the surface, remains synthetically challenging when compared to planar or spherical surfaces [17,27]. This is particularly true for pores less than 10 nm in diameter [17]. Polymer-functionalised pores below 10 nm generally become inaccessible, and the grafted polymer is generally multimodal with a broad molecular weight distribution [17].

Surface-initiated atom transfer radical polymerisation (SI-ATRP) remains the most widely investigated technique due to its wide range of polymerisable monomers with controllable molecular weight and narrow dispersities, chemical tolerance, and mild polymerisation conditions [3,18]. While several techniques such as initiators for continuous activator regeneration (ICAR) [3] and activators (re)generated by electron transfer (A(R)GET) [10] have been implemented to reduce the concentration of the copper catalyst that contaminates the final material, ATRP systems are more onerous, requiring numerous components to mediate a controlled polymerisation. This could severely limit the pore size that can be functionalised in terms of the control achievable and the polymer content.

Photoinitiated systems such as photoiniferter-mediated polymerisation (PIMP) are becoming increasingly popular in material functionalisation [18,23,24,28]. PIMP is advantageous in its synthetic simplicity, requiring minimal reaction components and proceeding by the simple application of UV irradiation. PIMP is also versatile, being effectively applied in surface functionalisation with a number of functional monomers including styrene [29], methyl methacrylate (MMA) [10,29], 2-(dimethylamino)ethyl methacrylate (DMAEMA) [30], [2-(methacryloyloxy)ethyl]trimethylammonium chloride (METAC) [24], and zwitterionic monomers [19,30]. Surface-initiated polymerisation (SIP) activated by UV irradiation also permits spatial and temporal control over the surface functionalisation to specify the location, degree of functionalisation, and even allowing surface patterning [18,23,28,30]. For a more detailed overview of the various SIP techniques applied to functionalise surfaces and the impact of surface curvature, the reader is referred to a recent and comprehensive review by Klok and co-workers [18].

While it is possible to functionalise small pores below 10 nm via SI-CLRP techniques, it is rather challenging to control the polymerisation kinetics, resulting in poorly accessible pores [17]. Consequently, selective functionalisation of the external surface has taken precedence in many studies. For instance, no current reports have attempted the formation of block copolymers within mesoporous materials. Although several reports have incorporated block copolymers selectively to the exterior surface of mesoporous silica nanoparticles for gating and controlled drug release [15,31]. To design and fabricate well-controlled uniform and responsive polymer coatings under the confines of mesopores below 10 nm, it is essential to understand how confinement impacts the polymerisation kinetics. Our research group previously reported an iniferter-initiated polymerisation of a zwitterionic monomer, carboxybetaine methacrylate (CBMA), from the interior and exterior surface of mesoporous silica with pores ranging from 2 to 115 nm in diameter, focussing on the effect of pore size, grafting density, and initiator on the SIP from iniferters that were post-grafted to the mesoporous silica surface through a terminal trimethoxysilane moiety [24]. In this system, a certain degree of control over the polymer content was achieved; however, the pore filling ratio was limited under the conditions used, and the post-grafting strategy of the iniferter does not permit localised polymer functionalisation.

**Scheme 1:**
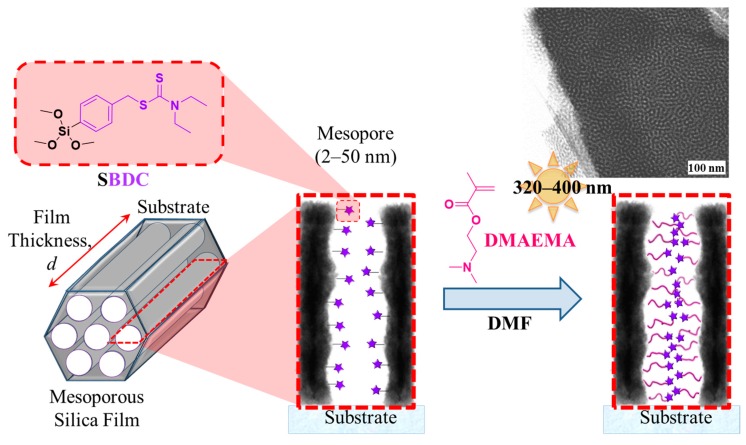
Mesoporous silica thin films functionalised with an organosilane bearing a terminal trimethoxysilane (*N*,*N*-(diethylamino)dithiocarbamoyl-benzyl(trimethoxy)silane, SBDC) can be easily functionalised in the presence of a functional monomer, UV light, and solvent (*N*,*N*-dimethylformamide, DMF).

Herein, we report a simple one-step approach to functionalise mesoporous silica films with a responsive polymer through surface-initiated photoiniferter-mediated living radical polymerisation (SI-PIMP). We further explore the effect of confinement on the polymerisation kinetics of 2-(dimethylamino)ethyl methacrylate (DMAEMA), and its impact on the amount of polymer formed within the mesopores, including the use and effect of a sacrificial iniferter on film functionalisation, and how this impacts the wetting and ionic permselectivity behaviour of the hybrid materials. Our approach involves the incorporation of a photoiniferter, *N*,*N*-(diethylamino)dithiocarbamoyl-benzyl(trimethoxy)silane) (SBDC), in a one-pot procedure through co-condensation with an inorganic precursor combining sol–gel chemistry and evaporation-induced self-assembly (EISA) to prepare a functional mesoporous thin film through dip-coating that is capable of undergoing polymerisation on exposure to UV light in the presence of a monomer as depicted in Scheme 1. We will further demonstrate that the formation of block copolymers is possible under confinement.

## 2. Materials and Methods

### 2.1. Materials

All materials and solvents were purchased from Sigma-Aldrich (Darmstadt, Germany) and used as received unless stated otherwise. The impact of a sacrificial iniferter was examined by the addition of benzyl diethyldithiocarbamate (BDC), a structural mimic of *N*,*N*-(diethylamino)dithiocarbamoyl-benzyl(trimethoxy)silane) (SBDC), which were synthesised according to a previous literature protocol [29]. The monomer used to study the photoiniferter polymerisations from BDC and/or SBDC, 2-(dimethylamino)ethyl methacrylate (DMAEMA, 98%) was passed over neutral alumina immediately prior to use to remove the hydroquinone monomethyl ether (MEHQ) inhibitor, and the solvent *N*,*N*-dimethylformamide (DMF, anhydrous, 99.8%) was used as received. For re-initiation studies, 2-(methacryloyloxy)ethyl phosphate (MEP) was used as received.

The precursor solutions for dip-coating mesoporous films were prepared using the inorganic precursor tetraethoxysilane (TEOS, Acros Organics, 98%, Geel, Belgium) alone or in combination with an organosilane such as SBDC, in dry tetrahydrofuran (THF, Merck, SeccoSolv, Darmstadt, Germany) or absolute ethanol (EtOH, Merck, ≥99.5%), with a hydrochloric acid catalyst (HCl, 37%) and Milli-Q water. Pluronic^®^ F-127 (F127, BioReagent, Sigma-Aldrich, 13,800 g mol^−1^) was used as a structuring agent in the sol, which was used as received at a molar ratio of 0.0050 or 0.0075 relative to the silane precursor to control the porosity of the mesoporous films prepared. The microscope slides (VWR, glass, cut edges) were cleaned in a mixture of detergent and water prior to rinsing with ethanol and drying under ambient conditions. The silicon wafers (Si-Mat, Kaufering, Germany, 100 mm diameter, 525 ± 25 µm thickness, type P/Bor, <100> orientation, CZ growth method, 2–5 Ω resistivity, polished on 1 side) and indium tin oxide (ITO, Delta Technologies, Ltd., Loveland, CO, USA, polished float glass, 150 × 150 × 1.1 mm, SiO_2_ passivated/Indium Tin Oxide coated one surface, *R*_S_ = 4–8 Ω, cut edges) were cut to an appropriate size using a diamond cutter, cleaned using technical grade ethanol, and dried under ambient conditions prior to dip-coating.

### 2.2. Characterisation

#### 2.2.1. Ellipsometry

Ellipsometry measurements of mesoporous films prepared on silicon wafer substrates (Si-Mat, Kaufering, Germany) were obtained from at least three spots per film to determine the average film thickness and refractive index using a Nanofilm EP3 imaging ellipsometer equipped with a 658 nm laser. Measurements were recorded at a fixed humidity of 15% at room temperature, and the angle of incidence (AOI) was varied from 38° to 66° in one zone in 2° increments. The EP4 analysis software supplied with the instrument was used to calculate the film thickness and refractive index from the measured amplitude and phase difference, Ψ and Δ, respectively. The data was fit using a one-layer box model, assuming an oxide layer on the silicon wafer was present between the substrate and mesoporous film, and this was fixed at 2.2 nm from previous measurements and fitting. The film thickness was allowed to vary between 10 and 300 nm, with a refractive index between 1 and 1.7. The Brüggemann effective medium approximation was used to calculate the volume porosity from the fitted refractive index [32,33]. Detailed calculations are provided in the Supplementary Materials (Section 5, Scheme S1).

#### 2.2.2. Cyclic Voltammetry (CV)

The electrochemical and transport properties of the mesoporous materials was investigated via cyclic voltammetry using $\mathrm{Fe}{\left(\mathrm{CN}\right)}_{6}^{4-/3-}$ and $\mathrm{Ru}{\left({\mathrm{NH}}_{3}\right)}_{6}^{2+/3+}$ as negative and positive probe molecules, respectively. Measurements were recorded using a Metrohm Autolab PGSTAT302N potentiostat (Metrohm, Utrecht, The Netherlands). Mesoporous films prepared on ITO substrates were characterised using a 2 mM solution of either the positive or negative probe molecule in a 100 mM KCl electrolyte solution. The pH-dependent permselectivity was investigated by adjusting the solution pH between 2 and 11 by the addition of either aqueous NaOH or HCl to the prepared solutions. The pH was determined using pH-Fix 0–14 indicator sticks (Laborbedarf, Article No. 0549, Carl Roth, Karlsruhe, Germany). A Ag/AgCl reference electrode (BASi RE-6) and graphite counter electrode was used in the sample cell. The electrode area was 0.21 cm^2^; however, all measurements reported here correspond to the peak currents in μA. Each pH was measured using a scan rate sequence of 200, 100, 25, 300, 1000, and 200 mV s^‒1^, with each scan rate cycled three times. All voltammograms shown here were measured at 100 mV s^‒1^ from the last cycle recorded, and bare ITO was measured as a reference on each measurement day for comparison.

#### 2.2.3. Static Contact Angle (CA)

CA measurements of the prepared films were measured using a DataPhysics OCA35 instrument (DataPhysics Instruments GmbH, Filderstadt, Germany) and evaluated using SCA20 software (Version 4.5.15 build 1064, DataPhysics Instruments GmbH, Filderstadt, Germany, 1998–2013) through the sessile drop method. All measurements were conducted within a climate-controlled room with a temperature of 25 °C and a relative humidity of 50%. Contact angles were determined within 30 s of applying a 2 µL water droplet onto the surface at a rate of 2 µL s^‒1^. All values reported arise from averaging at least 3–5 measurements across each film surface, and are reported with a standard deviation. Before each film was measured, it was stored under the measurement conditions within the climate-controlled room for several hours or overnight.

#### 2.2.4. Attenuated Total Reflectance Fourier Transform Infrared Spectroscopy (ATR-FTIR)

Infrared spectra of the prepared mesoporous films on glass or ITO substrates were performed using a Perkin Elmer Instrument One Spectrum FT-IR Spectrometer equipped with a Universal ATR Polarization Accessory (Waltham, MA, USA). All spectra shown were averaged from at least 3 spots and normalised to the Si–O–Si band at ~1085 cm^‒1^. The spectra were recorded using the Spectrum Software (Version 10.5.4.738, PerkinElmer, Inc., Waltham, MA, USA, 2016) between 4000 and 650 cm^−1^ with a resolution of 4 cm^−1^, and a background correction was automatically applied. All further data corrections were performed in OriginPro9 (ADDITIVE Soft- und Hardware für Technik und Wissenschaft GmbH, Friedrichsdorf, Germany, 2012).

#### 2.2.5. Transmission Electron Microscopy (TEM)

Electron micrographs were recorded using a Philips FEI CM-20 transmission electron microscope (Philips, Amsterdam, The Netherlands) equipped with a LAB-6 cathode and Olympus CCD camera, and with a maximum resolution of 2.3 Å operating at an accelerating voltage of 200 kV. Samples were prepared by scratching the mesoporous films off the substrate and dispersing in filtered ethanol with 10 min of sonication before being drop cast onto 3.05 mm Cu grids (mesh size 200) with a Lacey carbon film (Plano GmbH, article number S166-2). The samples were then dried under ambient conditions.

#### 2.2.6. Scanning Electron Microscopy (SEM)

SEM spectra were obtained using a Philips XL30 FEG scanning electron microscope equipped with a tungsten cathode and a back scattered electron yttrium aluminium garnet (BSE YAG) detector with an accelerating voltage of 15–25 kV, a 30 µm aperture, and a spot size of 4–5. The samples were sputter-coated with a 5 nm coating of Pt/Pd. The digital micrographs were recorded over a range of magnifications at a working distance of approximately 10 nm using an SE2 detector.

#### 2.2.7. ^1^*H* Nuclear Magnetic Resonance (^1^*H* NMR) Spectroscopy

^1^*H* NMR spectra were recorded on a 300 MHz Bruker AVANCE NMR spectrometer (Billerica, MA, USA) in deuterated chloroform (CDCl_3_, 99.8% D), methanol (MeOD-*d*_4_, 99.8% D) or dimethyl sulfoxide (DMSO-*d*_6_, 99.8% D) unless specified otherwise. The solvent residual peak was used as an internal reference.

#### 2.2.8. Size Exclusion Chromatography (SEC)

SEC (Agilent Scientific Instruments, Santa Clara, CA, USA) was performed using a PSS-Agilent 1200 equipped with a 1200 Agilent RID and an autosampler (100 µL). The filtered samples were passed through a GRAM 10 µm pre-column (8 × 50 mm) and a PSS GRAM LINEAR 10 µm column (8 × 300 mm) using *N*,*N*-dimethylformamide (DMF) with 3 g L^‒1^ LiCl at a flow rate of 0.5 mL min^‒1^ at 25 °C. The molecular weight and molecular weight distribution was determined using WinGPC UniChrom software (Version 8.20 build 4815, PSS Polymer Standards Service GmbH, Mainz, Germay, 1992–2014) by conventional calibration with linear poly(methyl methacrylate) standards ranging between 2.2 × 10^2^ and 1.6 × 10^6^ g·mol^‒1^.

#### 2.2.9. Krypton BET Adsorption

Krypton sorption and desorption measurements were performed at 77 K using an Autosorb iQ2 from Quantachrome. The samples were degassed for 12 h prior to measurement, and the BET surface area was calculated in the relative pressure range of 0.1 to 0.3.

### 2.3. Experimental

#### 2.3.1. Synthesis of *N*,*N*-(Diethylamino)dithiocarbamoyl-benzyl(trimethoxy)silane) (SBDC), a Silane Terminated Iniferter

Sodium *N*,*N*-diethyldithiocarbamate trihydrate (STC) was dissolved in an excess of hot methanol to remove insoluble impurities. The solution was then dried over molecular sieves before being concentrated on a rotary evaporator and dried under high vacuum to remove any water or residual solvent. The purified sodium *N*,*N*-diethyldithiocarbamate (1.51 g, 8.81 mmol) was dissolved in dry THF (10 mL) before being added dropwise via syringe to a solution of *p*-(chloromethyl)-phenyltrimethoxysilane (1.93 mL, 8.76 mmol) in dry THF (10 mL) under a nitrogen atmosphere. The solution was then stirred at room temperature for 4 h before being filtered to remove the sodium chloride and dried under reduced pressure to remove the THF to yield crude SDBC (2) as a pale yellow liquid (76% crude yield). The crude product was then vacuum distilled at 160 °C using a Kugelrohr to yield pure SBDC as a yellow liquid (64% yield). ^1^*H* NMR (300 MHz, CDCl_3_, ppm): δ 7.53 (d, 2H, C_6_H_4_), 7.35 (d, 2H, C_6_H_4_), 4.49 (s, 2H, –C*H*_2_S–), 3.98 (q, 2H, –NC*H*_2_–), 3.66 (q, 2H, –NC*H*_2_–), 3.55 (s, 9H, –Si(OC*H*_3_)_3_), 1.22 (t, 6H, –NCH_2_C*H*_3_). MS (ES)^+^: *m*/*z* (%) calcd. for C_15_H_25_O_3_NS_2_Si [M^∙^]^+^ 359, found 359 (100) [M^∙^]^+^, 360 (23) [M + 1]^+^, 361 (16) [M + 2]^+^, 362 (4) [M + 3]^+^, 363 (1) [M + 4]^+^. Assigned NMR and MS spectra are provided in the Supplementary Materials (Figures S1 and S4).

#### 2.3.2. Synthesis of Benzyl Diethyldithiocarbamate (BDC), a Sacrificial Iniferter and SBDC Mimic

Recrystallised *N*,*N*-diethyldithiocarbamate (1.50 g, 8.77 mmol) was dissolved in dry THF (10 mL). This solution was then added dropwise via syringe to a solution of benzyl chloride (1105 μL, 9.62 mmol) in dry THF (10 mL) under a nitrogen atmosphere. The solution was then stirred for 3 h before being filtered to remove the sodium chloride. The filtrate was then dried under reduced pressure to remove the THF to yield crude BDC as a dark yellow-brown liquid (66% yield). The crude product was then vacuum distilled at 60–100 16C at 5 × 10^−1^ mbar using a Kugelrohr. ^1^*H* NMR (300 MHz, CDCl_3_, ppm): δ 7.33–7.16 (m, 5H, C_6_*H*_5_), 4.47 (s, 2H, –SC*H*_2_Ph), 3.97 (q, 2H, –NC*H*_2_CH_3_), 3.65 (q, 2H, –NC*H*_2_CH_3_), 1.21 (t, 6H, –NCH_2_C*H*_3_). MS (ES)^+^: *m*/*z* (%) calcd. for C_15_H_25_O_3_NS_2_Si [M^∙^]^+^ 239, found 239 (100) [M^∙^]^+^, 240 (16) [M + 1]^+^, 241 (10) [M + 2]^+^, 242 (1) [M + 3]^+^. Assigned NMR and MS spectra are provided in the Supplementary Materials (Figures S2, S3 and S5).

#### 2.3.3. Preparation of Mesoporous Silica Thin Films

The mesoporous silica films were prepared via sol–gel chemistry using tetraethoxysilane (TEOS) as an inorganic precursor either alone or in combination with a functional organosilane (SBDC) in order to perform a photo-initiated polymerisation. The sol contained an amphiphilic triblock copolymer, F127, which undergoes micellisation upon solvent evaporation resulting in the formation of a porous inorganic network. The precursor solutions were stirred for 24 h under ambient conditions before being used to prepare films through evaporation-induced self-assembly (EISA). Dip-coating was performed in a climate-controlled chamber at a temperature of 25 °C and a relative humidity of 50%, at a withdrawal speed of 2 mm s^−1^ unless stated otherwise. The precursor solution used to prepare mesoporous silica with 5 mol % SBDC (5SBDCSi) contained a final TEOS:SDBC:F127:THF:H_2_O:HCl molar ratio of 0.95:0.05:0.0075:20:5.2:0.28, and these films were prepared at a reduced temperature of 15 °C. After aging the films for 24 h under climate-controlled conditions, the films were subjected to a stabilising thermal treatment: 60 °C for 24 h, 130 °C for 24 h, and then finally at 200 °C for 1–2 h. The F127 template was then removed by immersion into a 0.01 M HCl ethanoic solution for 3 days with stirring. Detailed optimisation of the film preparation conditions including the withdrawal speed, temperature, and relative humidity are provided in the Supplementary Materials (Figures S8 and S9), as well as the full structural characterisation of the final 5SBDCSi films used in this study (Figure S10). 

#### 2.3.4. Surface-Initiated Photoiniferter-Mediated Polymerisation (SI-PIMP) from SBDC-Functionalised Mesoporous Silica

BDC (0.057 g, 0.24 mmol) and DMAEMA (1.0 mL, 5.9 mmol) were dissolved in DMF (8 mL), and the reaction mixture deoxygenated by nitrogen bubbling under light protection. The solution was then transferred via a deoxygenated syringe to a sealed flask containing the SBDC-functionalised mesoporous films on either glass, silicon wafer, or ITO substrates, which had been previously dried in vacuo, and deoxygenated using 3 vacuum–nitrogen cycles before being placed under a slight underpressure. The flask was then irradiated with UV light (320–400 nm) for various periods of time before exposure to air to quench the polymerisation. The free polymer formed was collected, dried under reduced pressure, and characterised by ^1^*H* NMR spectroscopy and SEC to determine the conversion and molecular weight, respectively. The films were extracted in THF at least 3 times to remove any physisorbed material before drying under ambient conditions for further characterisation. Unless otherwise stated, the DMAEMA:BDC molar ratio was maintained at 25:1, with a monomer to solvent ratio of 1:8 (*v*/*v*). Investigations in the absence of sacrificial iniferter were carried out in a similar fashion with an identical monomer concentration. A Lumatec UV-Technik Superlite 410 lamp was used as the light source in all photoiniferter polymerisations unless stated otherwise. The wavelength ranged from 320 to 400 nm (UVA) with an intensity of ~6 mW cm^‒2^ (see Supplementary Materials, Figure S6). Each polymerisation was carried out in a specially designed Schlenk flask with rectangular sides, and positioned ~10 cm away from the light source in an aluminium foil-lined box.

#### 2.3.5. CO_2_ Plasma Treatment

The organic functional groups present on the exterior surface of the mesoporous silica films were removed by applying a CO_2_ plasma treatment using a Diener Femto plasma cleaner (Diener electronic, Ebhausen, Germany) at a pressure of 0.4 mbar and power of 20% for 12 s according to a previously published protocol by Babu et al. [34].

## 3. Results and Discussion

### 3.1. Controlling a Confined Polymerisation

Successful and well-controlled polymerisation within a porous material depends on a number of additional factors when compared to a solution polymerisation: the accessibility of the reagents and pore blocking [18], initiator grafting density [35], exterior surface [24], surface curvature [18,19], and polymerisation time. To tune the amount of polymer incorporated within mesoporous materials and the level of control obtained—i.e., the molecular weight and corresponding molecular weight distribution of the surface-fixed polymers—these factors must be considered.

Photoiniferter-mediated polymerisation (PIMP) offers great potential to tune the surface properties, and hence the ionic permselectivity, of mesoporous films simply by exposure to UV light and appropriate monomers as depicted in Scheme 1 [22,28]. To achieve this, a functional organosilane (SBDC) bearing an iniferter group was co-condensed with TEOS in the presence of a templating agent (F127). The electron micrograph in Scheme 1 clearly shows a mesoporous structure formed with an approximate pore size of 8 nm. The polymerisable films have an average thickness of 232 nm and porosity of 21% from ellipsometry; and are characterised by a low specific surface area of approximately 4.9 m^2^/g according to BET measurements taken from substrate-supported films. Despite this low specific surface area arising from the incorporation of SBDC into the silica framework through co-condensation, the films display good permselectivity towards both positive and negative species (see Figure S10 and Table S1 in the Supplementary Materials for complete characterisation of the prepared films).

Co-condensation is an advantageous route over post-grafting onto a pre-formed mesoporous material; it ensures a uniform surface modification [7,36], and the successful co-condensation of an initiating group offers the potential to confine the initiating species to a specific layer in multilayer films for local polymer functionalisation. Previous approaches to functionalise mesoporous silica with functional polymers such as dual-responsive poly(2-(dimethylamino)ethyl methacrylate) (PDMAEMA) are labour-intensive involving multiple steps, and local polymer functionalisation is not conceivable [22,24]. This is the first report where an iniferter has been incorporated through co-condensation successfully.

The iniferter concentration was maintained at 5 mol % relative to TEOS to avoid significant disruptions during the self-assembly process [37,38,39]. Further details regarding the co-condensation conditions and the effect on the prepared material are provided in the Supplementary Materials (Section 3a). Furthermore, a low iniferter concentration minimises the radical concentration within the mesopores to suppress irreversible termination [24], as well as circumventing radical migration often observed in SI-RAFT-type systems that results in premature termination [40].

#### 3.1.1. Pore Accessibility

**Scheme 2:**
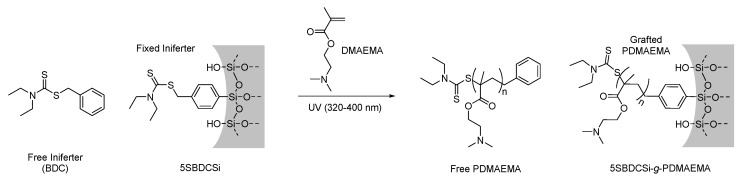
Proof of pore accessibility and the surface-initiated photoiniferter-mediated polymerisation (SI-PIMP) of 2-(dimethylamino)ethyl methacrylate (DMAEMA) from available *N*,*N*-(diethylamino)dithiocarbamoyl-benzyl(trimethoxy)silane (SBDC) surface groups within a mesoporous material in the presence of a sacrificial iniferter, benzyl diethyldithiocarbamate (BDC). Reaction conditions: [DMAEMA]:[BDC] = 25:1, DMAEMA:DMF = 1:8 (*v*/*v*), 4 h, 320–400 nm. Surface-initiated polymerisation (SIP) confined to pores by pre-treating the 5SBDCSi films with a CO_2_ plasma.

Our previous findings [24] suggest that slowing the polymerisation rate is key to the mediation of a controlled polymerisation under confinement; i.e., the radical concentration must be reduced to prevent premature termination due to the close proximity of radicals at concave surfaces. We employed several strategies to achieve this including a low concentration of surface-fixed iniferters, and high dilution. Consequently, the polymerisation was first studied in solution using a free or sacrificial iniferter (SI), BDC, which is structurally similar to SBDC (Scheme 2). The solution polymerisation proceeds in a controlled fashion using a monomer to initiator ratio of 25:1, and a monomer to solvent ratio of 1:8 (*v*/*v*) over a 4 h period under ambient temperature and constant irradiation with UV light (320–400 nm, 6 mW cm^‒2^). The solvent, *N*,*N*-dimethylformamide (DMF), was selected to solubilise the iniferter, monomer, and polymer in our initial kinetic studies. Under these conditions, a monomer conversion up to 60% is reached within 4 h with a corresponding dispersity exceeding 1.6 (see Supplementary Materials, Figure S7). These results are in agreement with general observations of iniferter-initiated polymerisations in which polymers synthesised in the presence of a photoiniferter are typically characterised by dispersities greater than 1.5, whilst dispersities of 1.1–1.4 are typical for their thermally-initiated counterparts [41].

The monomer and solvent must also be able to diffuse freely into the pores to access the initiating sites to successfully functionalise the inner pore walls of the mesoporous material. A CO_2_ plasma was applied prior to polymerisation to confirm that the SBDC initiating sites are accessible on the surface, and that the monomer can indeed access the mesopores and undergo polymerisation from the pore wall [34]. The optimised solution polymerisation was then applied directly to a mesoporous silica film co-condensed with 5 mol % SBDC relative to TEOS, the inorganic precursor, denoted 5SBDCSi (Scheme 2). After 4 h of irradiation, the free polymer formed in solution from the free iniferter reached a conversion of 58%, with a corresponding dispersity of 1.63.

**Figure 1:**
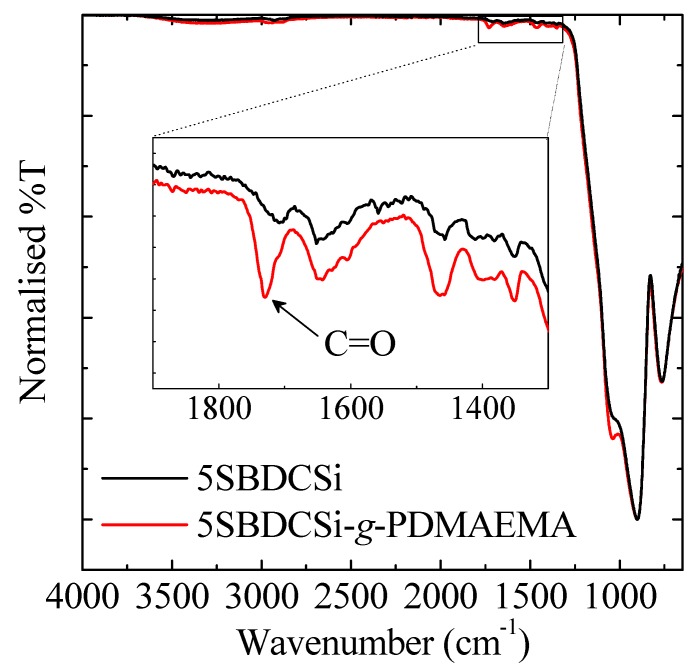
IR spectrum of a 5SBDCSi film on a glass substrate after performing a SI-PIMP of DMAEMA for 4 h in the presence of a SI (red trace) in comparison to a 5SBDCSi film on a glass substrate (black trace). The free polymer was characterised by ^1^*H* NMR (58% monomer conversion, *DP*_n,th_ 15, *M*_n,th_ 2520 g·mol^−1^) and SEC (*M*_n,SEC_ 3420 g·mol^−1^, *M*_w,SEC_ 5575 g·mol^−1^, *Ɖ* = 1.629). Reaction conditions: [DMAEMA]:[BDC] = 25:1, DMAEMA:DMF = 1:8 (*v*/*v*), 4 h, 320–400 nm.

**Figure 2:**
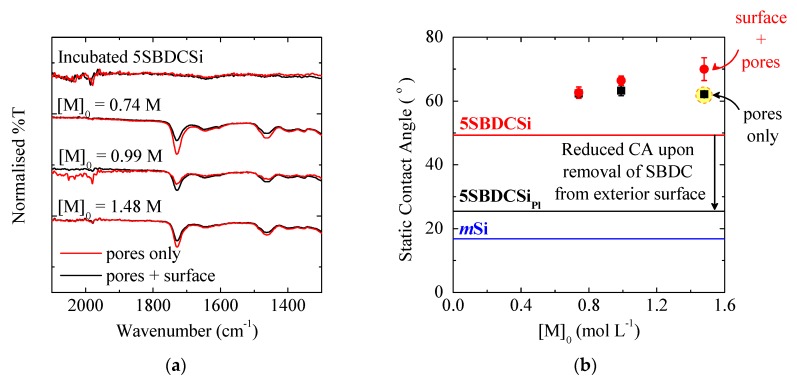
(**a**) IR; and (**b**) static CA measurements of mesoporous silica (*m*Si) co-condensed with 5 mol % SBDC with (5SBDCSi_Pl_) and without (5SBDCSi) a CO_2_ plasma treatment prior to irradiation with UV light for 4 h shows their successful functionalisation and potential to tune the polymer content by varying the initial monomer concentration, [M]_0_, from 0.74 to 0.99 and 1.48 M.

The appearance of a C=O stretching band at approximately 1730 cm^−1^ is associated with the formation of PDMAEMA on the mesopore surface (Figure 1). This observation indicates that the SBDC initiating groups are indeed present and available on the surface of the mesoporous framework. The free diffusion of monomer and solvent is further corroborated by incubation experiments (see Supplementary Materials, Figure S11). This is further supported by a decrease in the static contact angle of the 5SBDCSi film from ~45° to 27° (Figure 2b) upon performing a CO_2_ plasma treatment [19,29]. 

#### 3.1.2. Monomer Concentration

One strategy to enhance the amount of incorporated polymer is the pre-concentration of reagents within the pores prior to polymerisation by increasing the initial solution monomer concentration from 0.74 M. After extracting the residual monomer, low molecular weight polymer, and solvent in tetrahydrofuran (THF), the prepared materials were characterised by static contact angle (CA) measurements and infrared (IR) spectroscopy (Figure 2).

PDMAEMA was successfully grafted to the SBDC-fixed silica mesopores upon UV exposure with the appearance of a clear C=O band at approximately 1730 cm^−1^ in all IR spectra (Figure 2a). The absence of monomer and polymer peaks following incubation without irradiation confirms the solvent extraction conditions are sufficient, and verifies the polymerisation does not proceed in the absence of UV light.

There is no correlation between monomer concentration and polymer content by IR; however, CA measurements suggest that higher monomer concentrations lead to higher amounts of grafted PDMAEMA within the mesopores (Figure 2b). Faster polymerisation kinetics are expected under confinement [20,42]; the increased rate of polymerisation under confinement results in the observed higher amount of grafted polymer after the same polymerisation time when higher monomer concentrations are used. The disparity between the measured CA at a solution monomer concentration of 1.48 M compared to lower monomer concentrations, indicated in yellow, suggests irreversible termination is further exacerbated under concentrated conditions due to higher local radical concentrations. Whilst higher radical concentrations lead to premature termination within the pores, iniferter groups present on the exterior surface continue to grow over prolonged polymerisation times as evidenced by higher CAs (red circles), in agreement with our previous studies [24].

#### 3.1.3. Exterior Surface

While the exterior surface area is relatively small compared to the internal mesoporous structure, it can impact reagent diffusion to active sites within the pores as the polymer grows, resulting in the evolution of molecular weight gradients along the pore channels [19,20,35,42].

**Figure 3:**
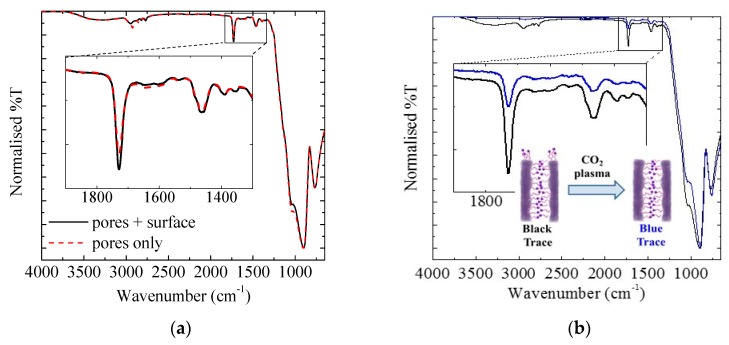
The presence of polymerisable groups and subsequent formation of a polymer on the exterior surface can hinder the formation of polymer within the mesopores: (**a**) IR spectra of 5SBDCSi after performing a SIP for 60 min where the SIP is confined exclusively to the pores (- - - -), and the pores and exterior surface (――); (**b**) IR spectra before (――) and after (――) performing an additional CO_2_ plasma treatment to remove the polymer formed on the exterior surface after polymerisation. Reaction conditions: DMAEMA:DMF = 1:8 (*v*/*v*), 1 h, 320–400 nm.

The SIP was therefore performed concurrently with and without a CO_2_ plasma pre-treatment to elucidate the impact of growing chains on the exterior surface on the polymer formed within the mesopores. The IR spectra are shown in Figure 3a. As expected, the amount of PDMAEMA formed is higher when the SBDC groups are present and undergo polymerisation from the exterior surface (――) than when confined exclusively within the mesopores (- - - -).

An additional CO_2_ plasma treatment was then applied to the polymer-functionalised mesoporous films to remove the polymer grafted from the exterior surface. A dramatic reduction in the C=O stretching band at ~1730 cm^−1^ (Figure 3b) clearly demonstrates that the functionalisation of the exterior surface dominates over the interior surface.

Furthermore, the amount of PDMAEMA incorporated within the mesopores is drastically lower when polymer grows simultaneously from the exterior surface and interior pore walls. This is clearly seen by comparing the insets in Figure 3a,b. When the polymerisation can proceed simultaneously inside the pores and from the exterior surface, i.e., no CO_2_ plasma pre-treatment prior to performing the SIP, the formation of polymer on the exterior surface severely hampers the SIP within the pores in the current system through monomer consumption and reduced diffusion of reagents. Hence, to achieve higher and more uniform polymer functionalisation, the polymerisation must be confined within the pores.

#### 3.1.4. Sacrificial Iniferters

Sacrificial initiators (SIs) are commonly used to control the surface-initiated polymerisation (SIP) from particles and planar substrates, particularly in SI-ATRP [19,43,44,45]. The free polymer is easily collected and characterised in place of the grafted polymer, and generally provides a good representation of the grafted chains. This assumption often circumvents the need for laborious and potentially hazardous degrafting of the surface-fixed chains [18,45,46].

Furthermore, as the free initiator is in excess of the surface-fixed initiators, the monomer to free initiator ratio dictates the polymerisation kinetics, and therefore the chain length formed on the surface. This allows the material properties to be precisely tuned, making the use of SIs an attractive tool in the preparation of organic‒inorganic hybrid materials with precisely targeted properties for a specific application. Currently, there are no exhaustive studies on the effect of sacrificial initiators under confinement. Studies which have incorporated sacrificial initiators under confinement have been limited to ATRP systems [19,20]. In these systems, the SI is required to ensure a sufficient quantity of deactivator is present to effectively control the polymerisation from the surface [18,19,20,21,44,46].

**Figure 4:**
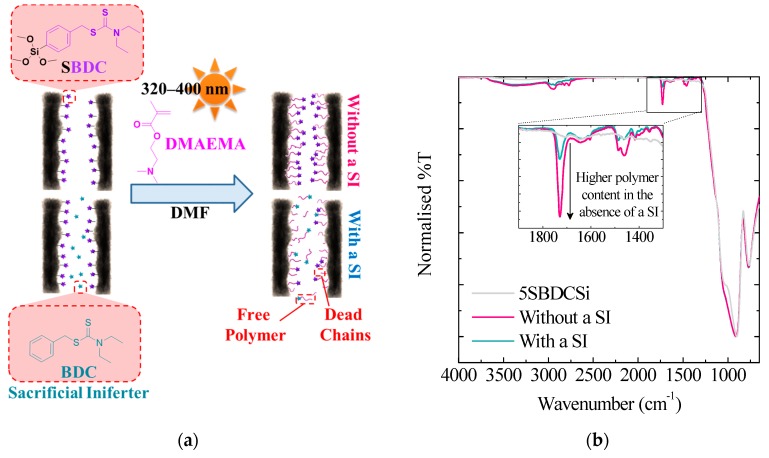
(**a**) Schematic diagram depicting the potential increased termination under confinement in the presence of a SI, BDC, leading to a reduced polymer amount as shown by (**b**) IR spectroscopy. Reaction conditions: DMAEMA:DMF = 1:8 (*v*/*v*), 4 h, 320–400 nm, and a [DMAEMA]:[BDC] molar ratio of 25:1 was used when performed in the presence of a SI (BDC).

In this system, the presence of a sacrificial iniferter, BDC, consistently results in a drastically lower polymer content within the mesopores. A representative IR spectrum is shown in Figure 4b, which is further supported by CA measurements (Table 1 and Tables S2 and S3). The effect of the added SI is more apparent when the SIP is confined within the mesopores, with a CA over 30° higher when performed in the absence of a SI of 86°. This observation further suggests that the formation of polymer on the exterior surface hinders reagent diffusion from solution into the mesopores, and that the polymerisation is accelerated under confinement. Benetti et al. [20] examined the confinement of SI-ATRP of methacrylates by adjusting the distance between a grafting surface and inert plane. They observed nearly a 4-fold increase in the brush molar mass when the SI-ATRP was performed under highly confined environments. The faster grafting kinetics under increasing confinement was attributed to the progressive hindering of the Cu(II) deactivator from the propagating chain ends. This effect was considerably more apparent in viscous media such as bulk polymerisations or in the presence of a SI, and contrary to non-confined SIPs. They also exploited this behaviour to prepared polymer gradients over a planar substrate by spatially tuning the vertical distance between the grafting and confining surface. However, this system varied between 400 nm and 10 µm.

It is well-documented that high SI concentrations lead to significantly lower molecular weight chains on the surface due to monomer consumption through the formation of free polymer in solution [47]. However, this is unlikely to play such an important role under the high dilutions used in this system. And contrary to ATRP and RAFT, each iniferter can initiate upon exposure to UV light leading to high local radical concentrations upon illumination. Consequently, the inclusion of a free photoiniferter creates a higher radical concentration within the mesopores; this may lead to increased irreversible termination in addition to higher monomer consumption. This scenario is depicted in Figure 4a.

**Figure 5:**
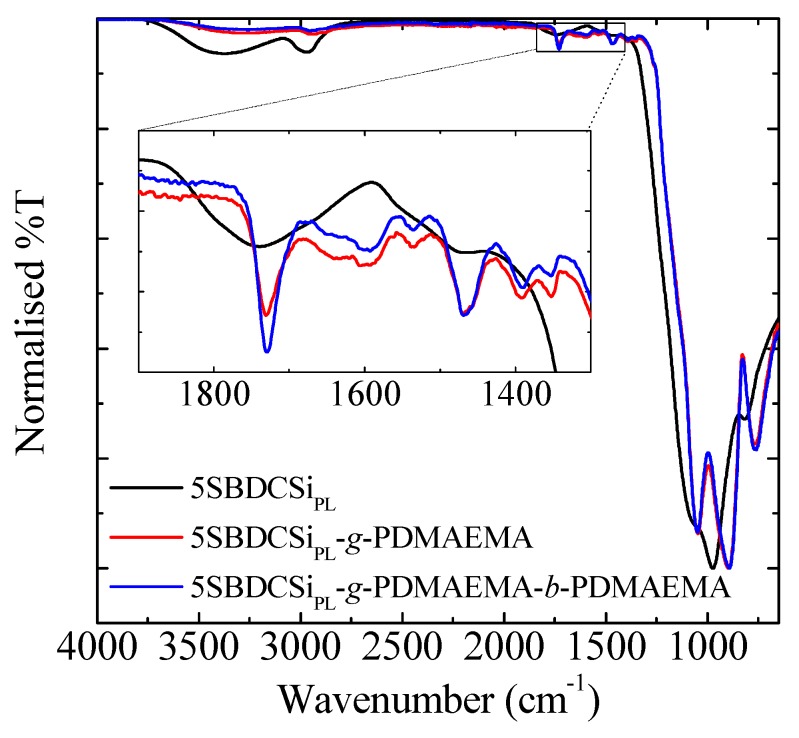
Normalised IR spectra of a CO_2_ plasma-treated 5SBDCSi film (black trace) in comparison to 5SBDCSi after performing a SIP in the presence of a SI for 30 min (red trace), followed by chain extension for a further 30 min (blue trace).

To elucidate the origin of the lower polymer content in the presence of BDC, successive SIPs were carried out to compare the livingness of the surface polymer formed in the presence and absence of BDC. The polymerisations were carried out in a similar fashion as previously described. Briefly, a mixture of DMAEMA (1 mL) in DMF (8 mL) was transferred to a flask containing two 5SBDCSi films under nitrogen protection before being irradiated with UV light. After 30 min, the polymerisation was quenched by exposure to air and one film was removed before the flask was sealed, deoxygenated, and then irradiated for a further 30 min. The films were then extracted with THF (3 × 30 min) to remove residual monomer, free polymer, and solvent. For comparison, this was also conducted in the presence of a SI, using a [DMAEMA]:[BDC] molar ratio of 25:1. The IR and CA results are summarised in Figure 5 and Table 2, respectively.

The increased intensity of the C=O stretching band at ~1730 cm^−1^ upon chain extension shown in Figure 5, in combination with the increase in static CA from 43° to 48° upon performing a second polymerisation in the presence of a SI, indicates that the chains possess living character and can re-initiate to form block co-polymers. Although the chain-end fidelity remains unknown from these investigations.

The rapid polymerisation rate in the absence of a SI, as evidenced from the high intensity C=O band (Figure 4b) and contact angles exceeding 60° that do not increase further upon performing a second polymerisation (Table 2), indicates the surface-fixed chains undergo irreversible termination. The low chain-end fidelity when performed in the absence of a SI likely arises from the rapid and uncontrolled polymerisation under confinement. These chains then terminate irreversibly in the absence of a sufficient quantity of stable radicals that a SI would provide to reversibly terminate growing chains upon exposure to air.

In this way, the presence of a SI may be beneficial in capturing growing radicals reversibly similarly to the reversible deactivator in ATRP. Hence, sacrificial iniferters offer the potential to mediate a controlled SIP with tuneable graft molecular weight under high dilution, and further permit the formation of block copolymers. The efficacy of PIMP to prepare block copolymers under confinement in the absence of a SI is further explored in Section 3.3.

#### 3.1.5. Polymerisation Time and SIP Kinetics

**Figure 6:**
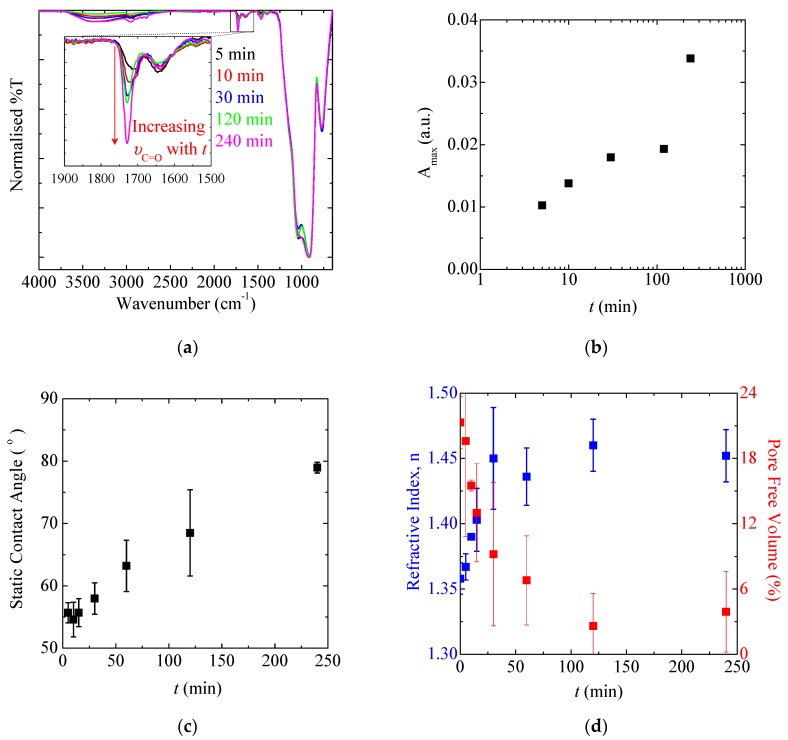
Polymerisation kinetics under confinement: (**a**) IR spectra; (**b**) corresponding C=O stretching absorbance associated with grafted PDMAEMA; (**c**) static CA measurements; and (**d**) refractive index (■) and corresponding free volume (■) from ellipsometry. Reaction conditions: DMAEMA:DMF = 1:8 (*v*/*v*), 0–240 min, SIP confined to pores by pre-treating the 5SBDCSi films with a CO_2_ plasma.

To improve our understanding of SI-PIMP kinetics under confinement in the absence of a sacrificial iniferter, 5SBDCSi films were pre-treated with a CO_2_ plasma to confine the polymerisation to the interior walls of the mesopores. This ensures the observed behaviour arises from the mesopore-bound polymer. Parallel polymerisations were then carried out for varying timescales between 5 and 240 min before being exposed to oxygen to quench the polymerisation and extracted in THF (3 × 10 min). The IR, CA, and ellipsometry results are summarised in Figure 6.

As shown in Figure 6a–c, the carbonyl stretching band and CA continue to increase over the irradiation time examined. This is a good indication that the polymerisation continues within the pores over the timescale explored in a controlled fashion. This is contrary to a previous system we reported where the SIP of CBMA within the pores was observed to cease after a relatively short time compared to the exterior surface, where the polymer grafts continue to grow for prolonged periods [24]. The premature demise of the SIP within the pores likely arises from the higher monomer concentration (see Section 3.1.2), surface curvature, and the initiator grafting density used. These factors lead to a higher local radical concentration within the mesopores. Electrostatic exclusion of the positively charged monomer from the polymer-functionalised mesopores may also be a limiting factor in that case. Moreover, using our optimised polymerisation conditions, it is possible to achieve high polymer fillings up to geometrical pore blocking within 60 min (Figure 6d), with a high degree of control over the polymer content (Figure 6a–c) regardless of whether a co-condensation or post-grafting strategy of SBDC is employed. The film stability under these conditions was also assessed to ensure pore blocking is indeed a consequence of grafted polymer (see Supplementary Materials, Figure S13).

When the current system proceeds without interference, i.e., without exposure to air (Section 3.1.4, Table 1), significantly higher contact angles nearing 80° are achievable after 240 min. In the absence of an added sacrificial initiator, the poor re-initiation observed upon chain extension supports our hypothesis that a high radical concentration is present that terminates irreversibly upon exposure to air, contrary to other controlled/“living” radical polymerisation techniques. In the case where a SI is present, the growing free polymers originating from the SI may further act as radical scavengers to produce dead free chains to allow further growth from initiating sites attached to the surface. The SI may also provide a sufficient concentration of stable radicals to reversibly deactivate the surface-fixed chains. This allows the surface polymer to continue to grow upon subsequent irradiation and exposure to a monomer.

Having demonstrated that the amount of polymer incorporated in a mesoporous silica film can be easily tuned by adjusting the polymerisation time and monomer concentration, we investigated the effect of polymer content on the ionic permselectivity behaviour.

### 3.2. Tuneable Ionic Permselectivity

**Figure 7:**
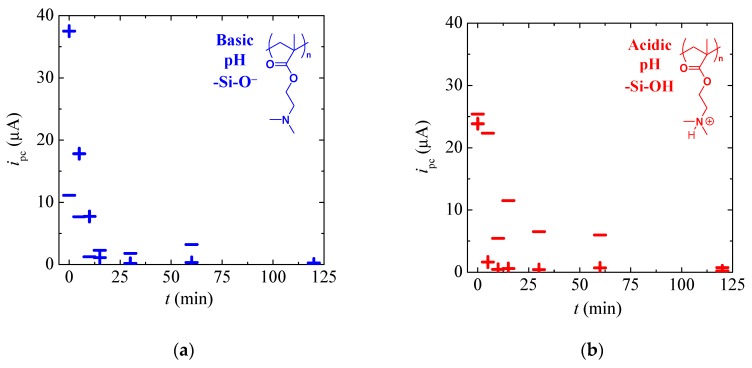
Ionic permselectivity with increasing polymerisation time under (**a**) basic and (**b**) acidic conditions for positive (+) and negative (–) probe molecules. Cathodic peak currents were recorded at a scan rate of 100 mV s^−1^. Reaction conditions: DMAEMA:DMF = 1:8 (*v*/*v*), 0–240 min, 320–400 nm, SIP confined to pores by pre-treating the 5SBDCSi films with a CO_2_ plasma.

The electrochemical properties of the organic–inorganic hybrid materials prepared via SI-PIMP were investigated by cyclic voltammetry (CV) using positive and negative probe molecules under acidic and basic conditions. The impact of the incorporated polymer is highlighted clearly by the measured peak currents, which are shown graphically with increasing polymerisation time in Figure 7. The raw CV traces are available in the Supplementary Materials for reference (Figure S12, Table S4).

The rapid reduction in peak current by over 99% under basic conditions towards positive probe molecules within 30 min correlates to a pore filling of ~70%, and supports rapid polymerisation and geometrical pore blocking, with gated transport towards both positive and negative species under basic conditions (Figure 7a). After approximately 25 min of polymerisation, there is a slight increase in the ionic pore accessibility towards negative charges. This may arise from the growing PDMAEMA chains shielding the negatively charged silica walls.

The significantly higher pore accessibility towards negatively-charged species under acidic conditions allows gated transport with amplitude control. The PDMAEMA-grafted mesoporous silica films are (i) permselective to anions under acidic conditions (pH < pK_a,PDMAEMA_); (ii) permselective to cations under basic conditions (pH > pK_a,PDMAEMA_); and (iii) gated towards cations at high polymer content regardless of pH.

There is a clear pore filling-pore wall charge effect that determines the observed permselectivity behaviour. The permselectivity correlates well with the pore filling calculated from ellipsometry measurements, which decreases rapidly with increased polymerisation time (Figure 6d, Table S4). The reduced wetting observed (Figure 6c) also impacts the permselectivity of aqueous solutions, and the contact angle increases approximately linearly with polymerisation time. Such behaviour is consistent with previous studies by Chen et al., [22] where the ionic permselectivity was investigated in mesoporous silica films grafted with PDMAEMA using SI-RAFT. They observed that, once the degree of polymerisation exceeded 75 monomer units, the conductivity decreased dramatically. This behaviour may be exacerbated by narrow necks relative to the pore diameter, and the significantly lower surface area of these co-condensed films when compared to pure mesoporous silica structured with F127 (Supplementary Materials, Table S1). It is worth noting that the polymer chain length is unknown. As these polymerisations were performed in the absence of a SI—which would theoretically limit the degree of polymerisation to 25 monomer units from an ideal surface, i.e., spherical or planar substrates—the polymerisation proceeds significantly faster under confinement compared to solution, and therefore the surface-bound polymer may in fact reach these high chain lengths to result in the observed pore blocking. To preserve the permselectivity of these films, it is important to avoid pore blocking, and incorporate a low polymer amount. Other approaches to reduce geometrical pore blocking resulting from termination and high polymerisation rates is slowing the polymerisation by further dilution and reducing the light intensity used to initiate the polymerisation [28]. However, studies at lower intensity and extended polymerisation times result in iniferter degradation [28].

**Scheme 3:**
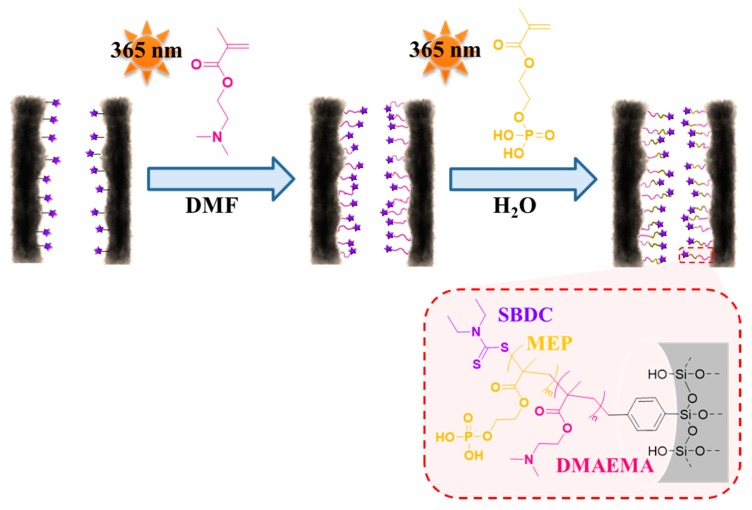
Schematic illustration of the re-initiation of PDMAEMA grafted from SBDC-functionalised mesoporous silica thin films to form block copolymers under confinement by exposure to UV light and appropriate monomers.

We have shown using DMAEMA as a model and dual-responsive monomer that it is possible to control the amount of polymer grafted from mesoporous silica films via SI-PIMP. This allows us to fine-tune the ionic permselectivity behaviour of these organic‒inorganic hybrid materials by controlling the charge density and/or wetting behaviour. To design materials with targeted properties for a specific application, the system should be easily adapted for other monomers, and even permit the formation of block copolymers (Scheme 3).

### 3.3. Re-Initiation and the Potential to form Block Copolymers under Confinement

**Figure 8:**
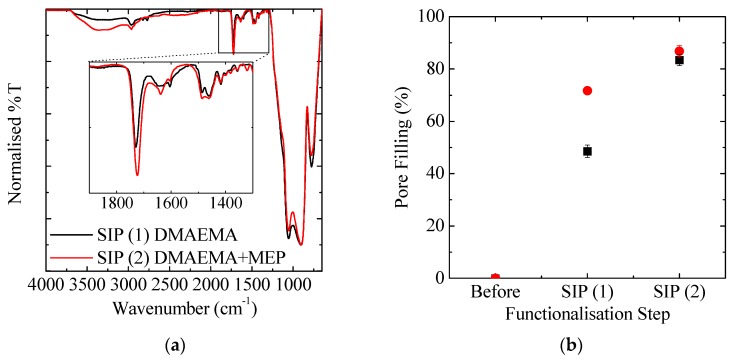
(**a**) IR spectra of mesoporous silica (16 nm pores) after performing a SIP with DMAEMA (SIP (1), black trace) and subsequent chain-extension with MEP (SIP (2), red trace); (**b**) corresponding volume pore filling of mesoporous silica with 8 nm (black squares) and 16 nm (red circles) pores post-grafted with SBDC before and after performing a SIP with DMAEMA (SIP (1)) and chain-extension with MEP (SIP (2)). The non-functionalised 8 and 16 nm mesoporous silica films are characterised by a porosity of 40 and 57%, respectively. Reaction conditions for SIP (1): DMAEMA:DMF = 1:8 (*v*/*v*), *t* = 5 min, λ = 365 nm. Reaction conditions for SIP (2): [MEP] = 0.125 M, solvent = water, *t* = 15 min, λ = 365 nm. The pore filling was estimated assuming the refractive index of the polymer is 1.517 (PDMAEMA) [49].

It is essential that we can control the polymer that forms from the surface in terms of molecular weight, dispersity, functionality, and architecture to design materials with specific and targeted properties. We have shown that it is possible to control the SIP under confinement by adjusting the monomer concentration, polymerisation time, and by the incorporation of a sacrificial iniferter. The ability to form block copolymers enables us to further tune the properties and behaviour of the hybrid materials. Due to higher levels of termination under confinement, particularly relevant in 8 nm pores, this is a considerable challenge to overcome. The incorporation of a SI also further complicates the polymerisation procedure, and requires more thorough washing to remove the free polymer from the hybrid material. Limiting the polymerisation time of the first block to 5 min under high dilution ensures high retention of active chain ends that can then be re-initiated upon exposure to a second monomer, 2-(methacryloyloxy)ethyl phosphate (MEP), under UV irradiation in the absence of a SI as shown in Figure 8. Under the applied conditions, re-initiation and probable chain extension is possible within both 8 and 16 nm mesopores [48], reaching a high pore filling irrespective of pore size. Re-initiation of the first block is the most probable scenario since the initiating sites located at the surface are less accessible due to sterics, particularly at the high pore filling after the formation of the first block. Further details and raw data are provided in the Supplementary Materials (Section 4, Figure S14, and Tables S5 and S6). 

## 4. Conclusions

The synthetic ease of preparing organic‒inorganic hybrid materials via SI-PIMP from a mesoporous silica support to pore blocking in pores less than 10 nm in diameter was demonstrated. The successful incorporation of an iniferter moiety, SBDC, through co-condensation provides a simple one-pot method to prepare functional mesoporous materials capable of further functionalisation by SIP simply by exposure to appropriate monomers and UV light. Moreover, co-condensation offers the potential to locally functionalise mesoporous materials, which is of high-importance in achieving fine control over ionic permselectivity with potential applications in self-sustained sensing and separation devices, including lab-on-chip devices. We will elaborate on this aspect in future reports.

We further demonstrated that the SIP from the inner surface is diffusion-limited, with significantly less polymer incorporated when polymer is able to grow from both the exterior and interior surface. This makes PIMP more suited for application under confinement when compared to ATRP since fewer reaction components are required to access the initiating sites, allowing the polymer content to be controlled in pores below 10 nm; PIMP also proceeds in the absence of any transition metal catalyst that ultimately pollutes the final material.

By the incorporation of a dual responsive polymer, PDMAEMA, we illustrated that the permselectivity towards both positively and negatively-charged species can be selectively controlled and fine-tuned by varying the polymer content and solution pH. We further showed that the formation of block copolymers under confinement is possible, a feat which has not yet been reported. The use of a sacrificial iniferter under high dilution was also investigated; sacrificial iniferters offer the possibility to control the molecular weight of the polymer grafted from the exterior surface, and potentially the amount of polymer incorporated within the pores in a controlled fashion by providing a sufficient concentration of stable radicals to deactivate surface-fixed polymer after prolonged polymerisation times. The amount of polymer incorporated within the pores can also be tuned by varying the initial monomer concentration, and polymerisation time.

## Figures and Tables

**Table 1:** Static CA measurements of the prepared 5SBDCSi substrates grafted with PDMAEMA ^1^.

Polymer Location	Static Contact Angle (°)		
	With BDC ^3^	Without BDC	Δ
Pores only ^2^	53 ± 1	86 ± 1	32
Pores + Exterior Surface	65 ± 2	79 ± 4	14

^1^ Reaction conditions: DMAEMA:DMF = 1:8 (*v*/*v*), 4 h, 320–400 nm; a [DMAEMA]:[BDC] molar ratio of 25:1 was used when performed in the presence of a SI. ^2^ The substrate was pre-treated with a CO_2_ plasma to remove the SBDC initiating sites from the exterior surface before performing the SIP. ^3^ The free polymer was characterised by NMR (62% monomer conversion, *DP*_n,th_ 15.6, *M*_n,th_ 2690 g mol^−1^) and SEC (*M*_n,SEC_ 2870 g mol^−1^, *M*_w,SEC_ 4790, *Ɖ* 1.67).

**Table 2:** Average static CA of a CO_2_ plasma-treated 5SBDCSi film before and after performing a SIP for 30 min, and then after a chain-extension for a further 30 min with and without a SI.

Total Polymerisation Time (min)	Static Contact Angle (°)	
	With a SI	Without a SI
0	27 ± 0	27 ± 0
30	43 ± 1	63 ± 3
60	48 ± 3	62 ± 3

## Supplementary Materials

Supplementary Materials is available online at www.mdpi.com/2073-4360/9/10/539/s1.
