# Supplementary material for: Optimisation of Surface-Initiated Photoiniferter-Mediated Polymerisation under Confinement, and the Formation of Block Copolymers in Mesoporous Films
Source: Polymers (Basel). 2017 Oct 23;9(10):539. doi: 10.3390/polym9100539 (PMC6418678; doi:10.3390/polym9100539)
Supplement: Supplementary file 1 [file polymers-09-00539-s001.pdf]

Supplementary Materials: Optimisation of surface-initiated photoiniferter-mediated polymerisation under confinement, and the formation of block copolymers in mesoporous films

Jessica C. Tom[1], Robert Brilmayer[1], Johannes Schmidt[2], and Annette Andrieu-Brunsen[1,*]

1. Structural Characterisation of the Prepared Iniferters, BDC and SBDC

1.1. NMR Spectra of the Prepared Iniferters

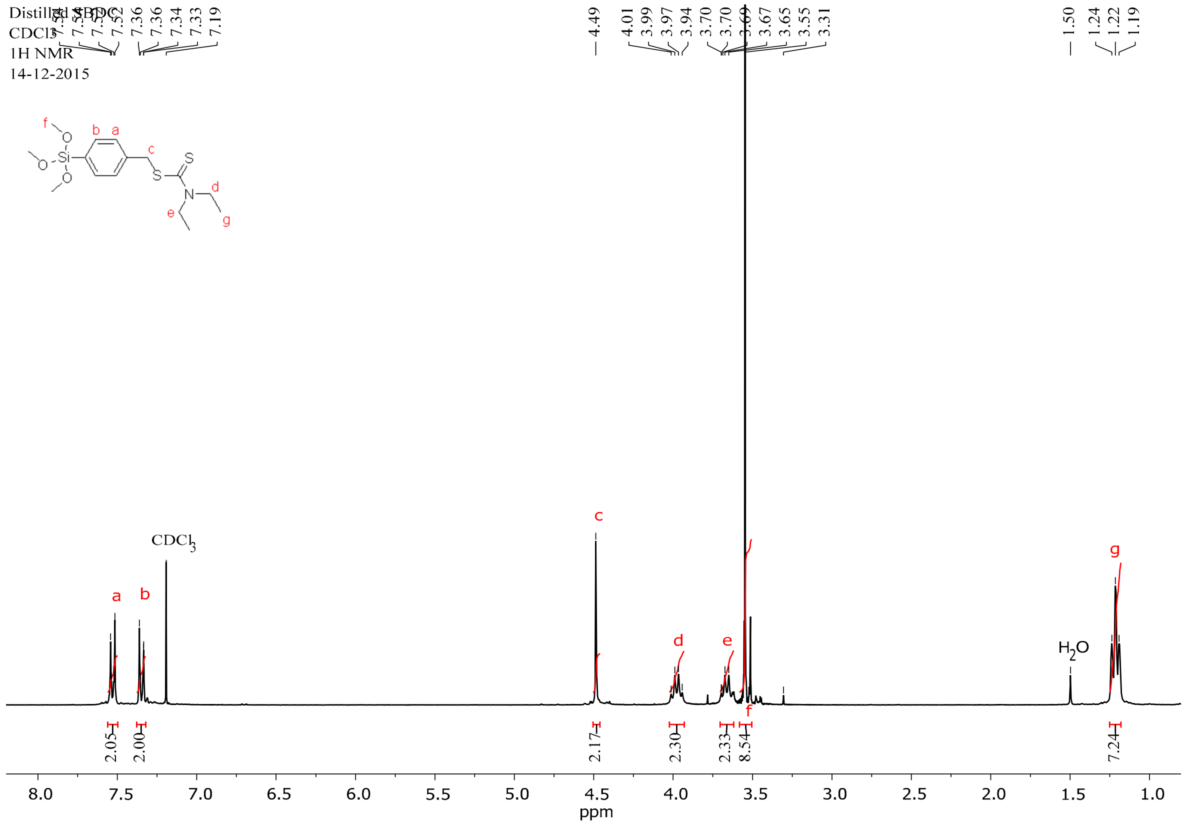

Figure S1. ^1H NMR spectrum of a synthesised organosilane, *N,N*-(diethylamino)dithiocarbamoyl-benzyl(trimethoxy)silane) (SBDC), after purification by distillation that can undergo polymerisation in the presence of UV light. The spectrum was recorded in $CDCl_3$ at 300 MHz. For further details, please refer to the Experimental, Section 2.3.1.

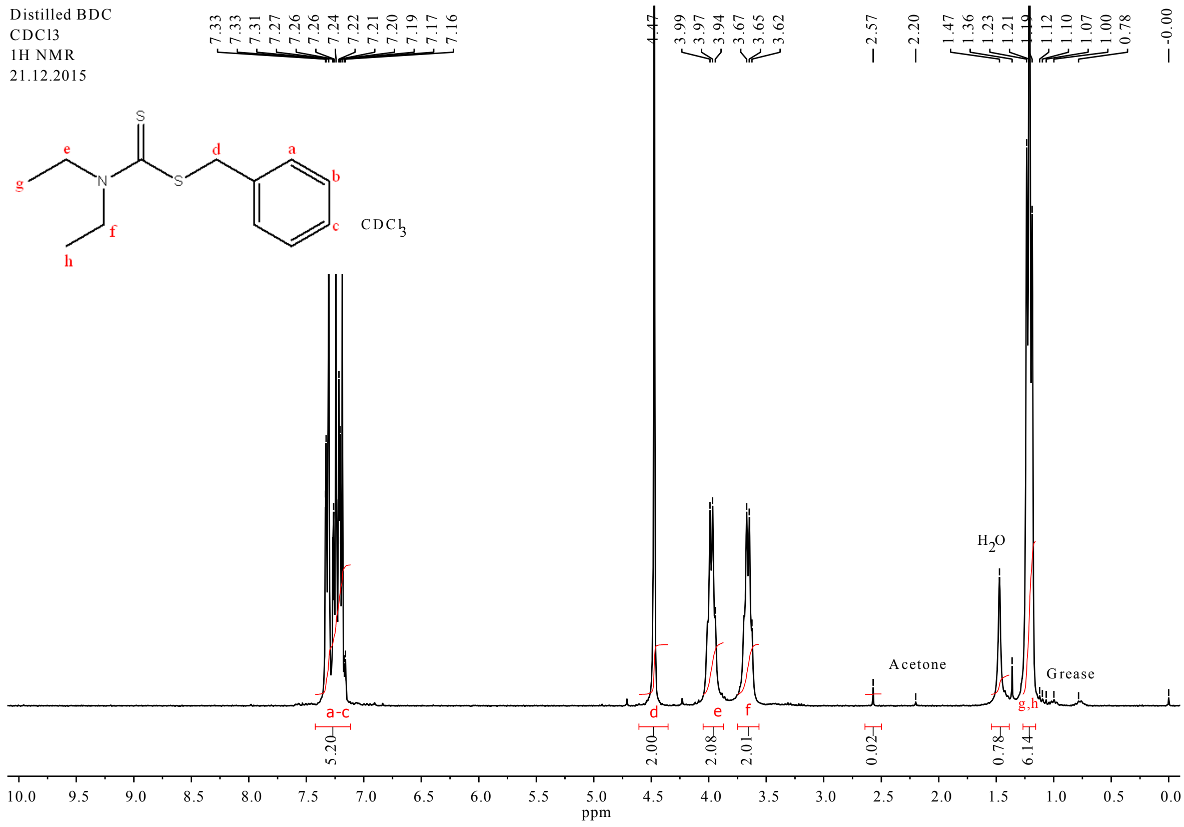

Figure S2. ^{1}H NMR spectrum of benzyl diethyldithiocarbamate (BDC) after purification by distillation. The spectrum was recorded in $CDCl_3$ at 300 MHz (Experimental, Section 2.3.2).

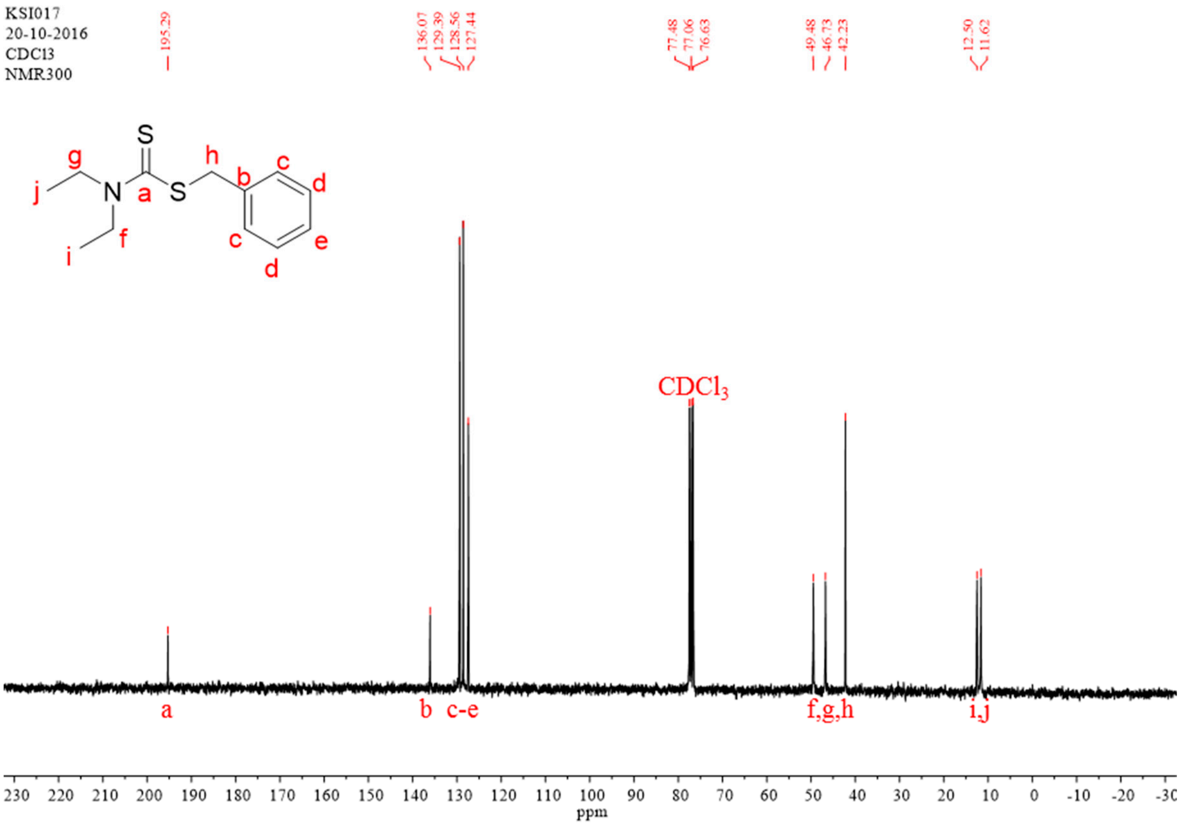

Figure S3. ^{13}C NMR spectrum of BDC after purification by distillation. The spectrum was recorded in $CDCl_3$ at 300 MHz (Experimental, Section 2.3.2).

1.2. Mass Spectra of the Prepared Iniferters

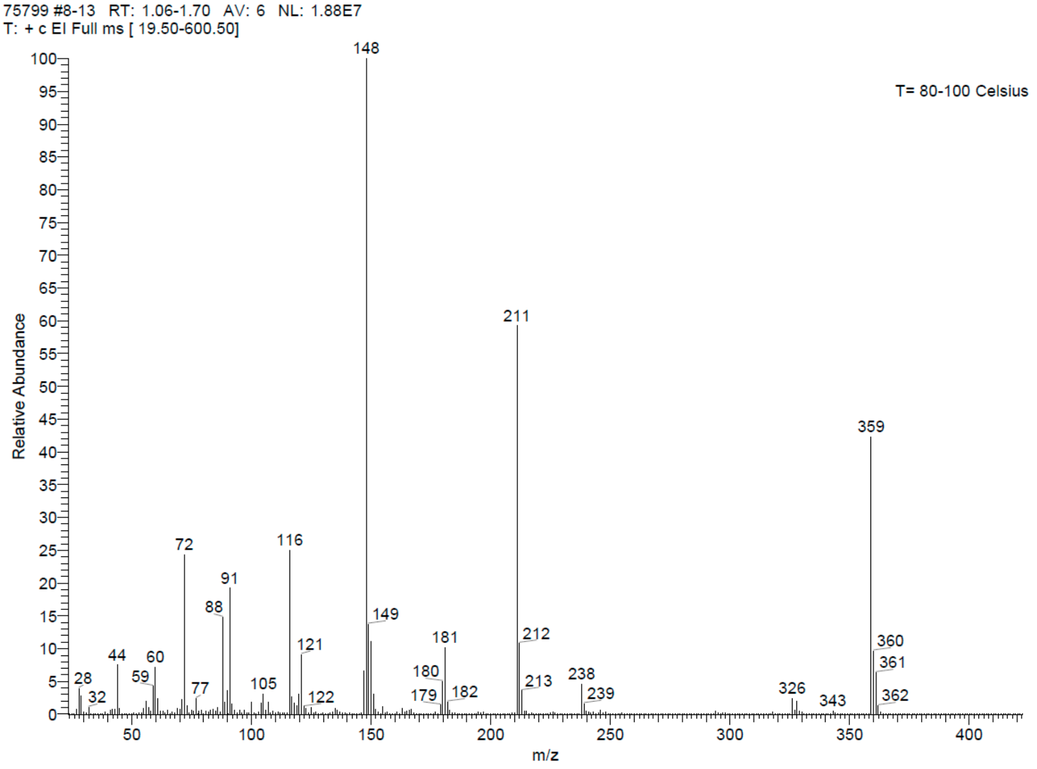

Figure S4. Electron ionisation mass spectrometry (EI-MS) of a synthesised organosilane, SBDC, after purification by distillation. m/z (%) calcd. for $C_{15}H_{25}O_3NS_2Si$ $[M^{\cdot}]^+$ 359, found 359 (100) $[M^{\cdot}]^+$, 360 (23) $[M+1]^+$, 361 (16) $[M+2]^+$, 362 (4) $[M+3]^+$, 363 (1) $[M+4]^+$ (Experimental, Section 2.3.1).

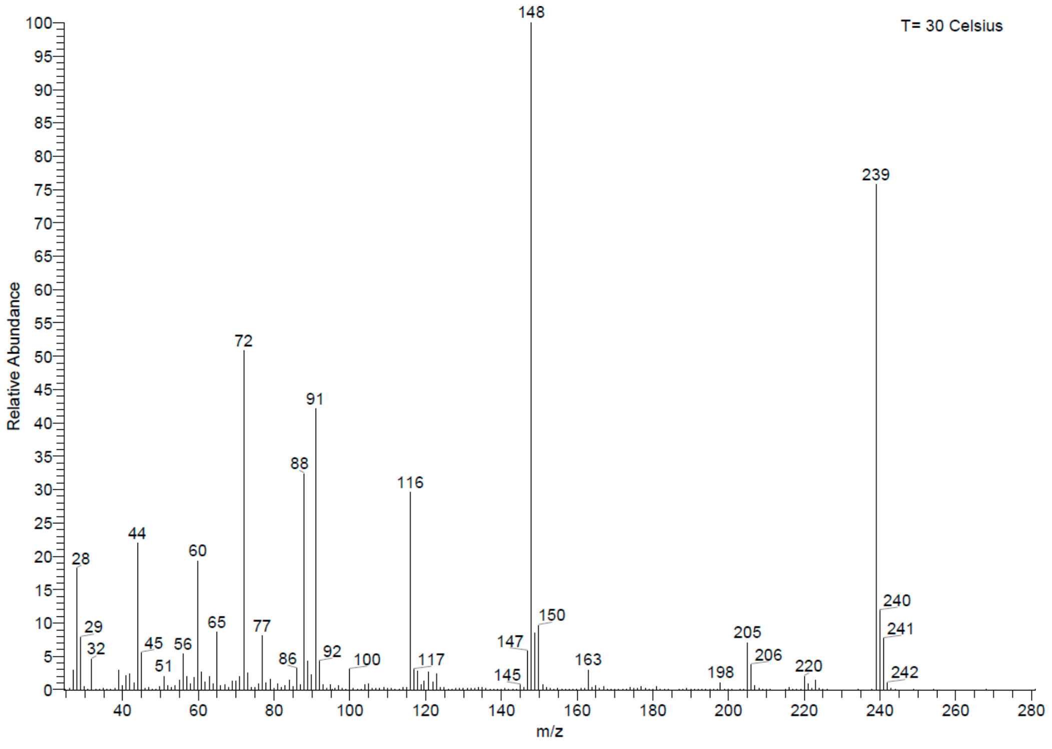

Figure S5. EI-MS of BDC after purification by distillation. m/z (%) calcd. for $C_{15}H_{25}O_3NS_2Si$ $[M^{\cdot}]^+$ 239, found 239 (100) $[M^{\cdot}]^+$, 240 (16) $[M+1]^+$, 241 (10) $[M+2]^+$, 242 (1) $[M+3]^+$ (Experimental, Section 2.3.2).

1.3. UV-Vis Absorption Spectra of the Prepared Iniferters in DMF in Comparison to the UV Lamp Spectral Profile

The polymerisation is initiated in the region of a local absorption minimum of both SBDC and BDC. Since the maximum absorption is below the DMF cut-off, it is the most appropriate wavelength range to initiate the polymerisation without changing the solvent. The solvent was chosen due to its ability to solubilise the iniferter, monomer, and polymer.

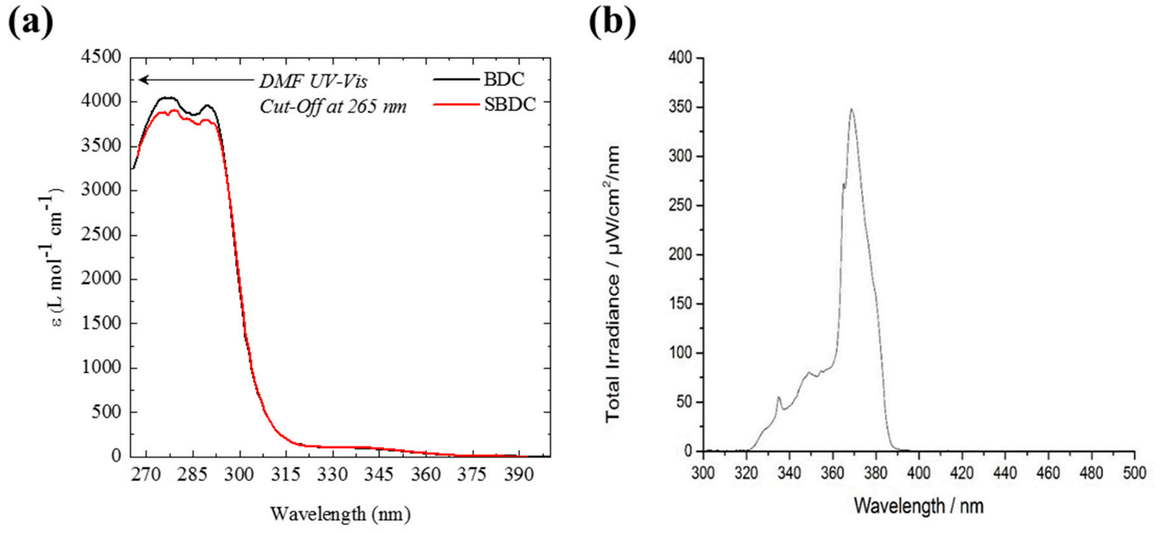

Figure S6. (**a**) UV-Vis absorption spectrum of BDC (black trace) and SBDC (red trace) in DMF, the polymerisation solvent, and (**b**) irradiation spectrum of the lumatec lamp used in all SI-PIMP investigations.

2. Solution Kinetics

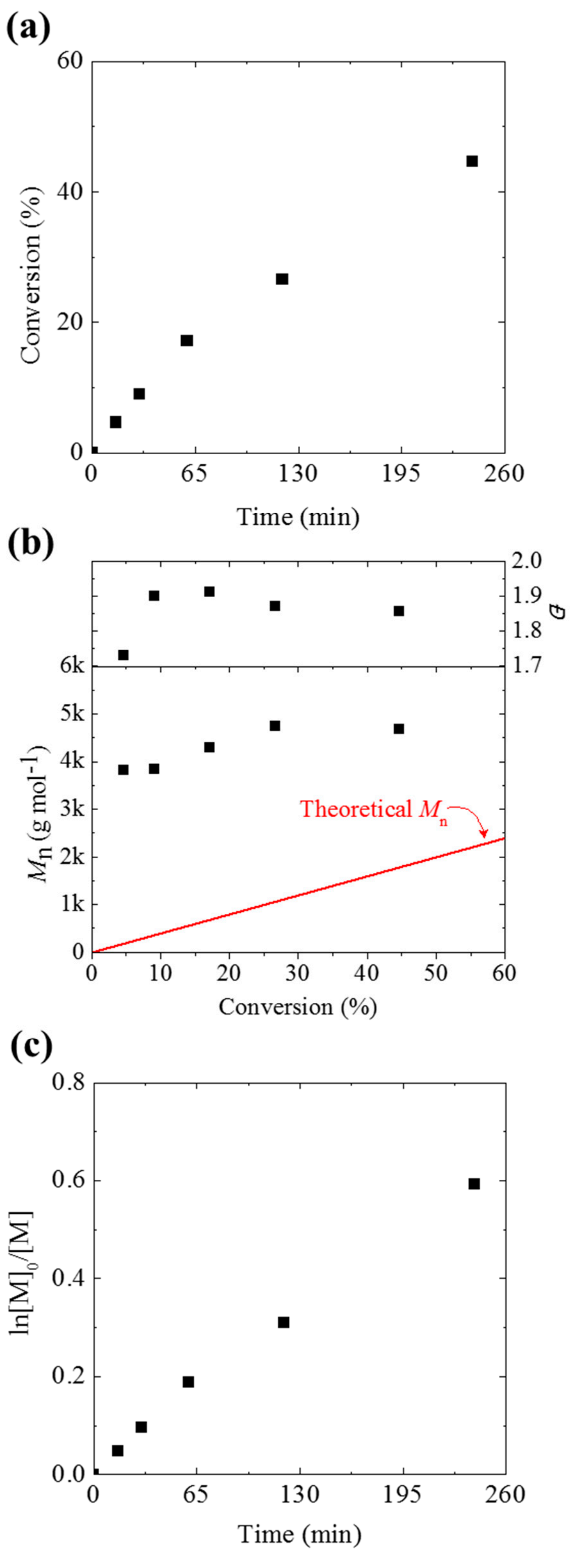

Figure S7. A typical solution PIMP of DMAEMA: kinetic plots showing (**a**) increasing conversion with time, (**b**) number average molecular weight and dispersity with increasing monomer conversion measured by SEC in DMF, and (**c**) semilogarithmic plot increasing linearly with conversion. Reaction conditions: [DMAEMA]:[BDC] = 25:1, DMAEMA:DMF = 1:8 (v/v), 4 h, 320–400 nm.

3. Surface-Initiated Photoiniferter-Mediated Polymerisation from Single Layer Films

3a. Optimisation of SBDC Co-Condensation for Subsequent Preparation of Step-Gradient Films for Localised Functionalisation by SI-PIMP

The successful incorporation of an iniferter function through co-condensation over post-grafting offers the potential for localised polymer functionalisation. Consequently, the co-condensation of the iniferter, SBDC, was first investigated. The iniferter must be available on the surface to undergo a SIP, and the mesopores must be interconnected to ensure a high permselectivity is retained.

While organosilanes are often employed in combination with inorganic precursors in sol–gel chemistry, due to the hydrophobicity and size of these compounds, it is often difficult to prepare mesoporous materials with high uniformity and order, as they can disrupt the assembly and condensation process [37–39]. To solubilise both SBDC and TEOS in a sol for dip-coating, tetrahydrofuran was used in place of ethanol. In addition, the concentration of SBDC was maintained at 5 mol% relative to TEOS to avoid any significant disruptions to the EISA process, and to maintain a low radical concentration within the pores to avoid excessive termination reactions.

Due to differences in solvent viscosity and volatility, the withdrawal speed, relative humidity and temperature at which the substrates were dip-coated was investigated. The materials were characterised by ellipsometry, CV, IR, SEM, and static CA measurements. The dip-coating withdrawal speed, v_{dip}, was first investigated: 1, 2, 4, and 8 mm s^{-1}. In comparison to pure mesoporous silica, which appear opalescent and quite transparent, these films had evident striations and variable drying lines. The intermediate withdrawal speeds of 2 and 4 mm s^{-1} resulted in a thickness of 294–295 nm, with a higher porosity of 32% achieved at a withdrawal speed of 2 mm s^{-1}. This film also displayed good permselectivity towards both positive and negative charges, and via IR appears to be the most promising in terms of the accessible surface functions. The results are summarised in Figure S8.

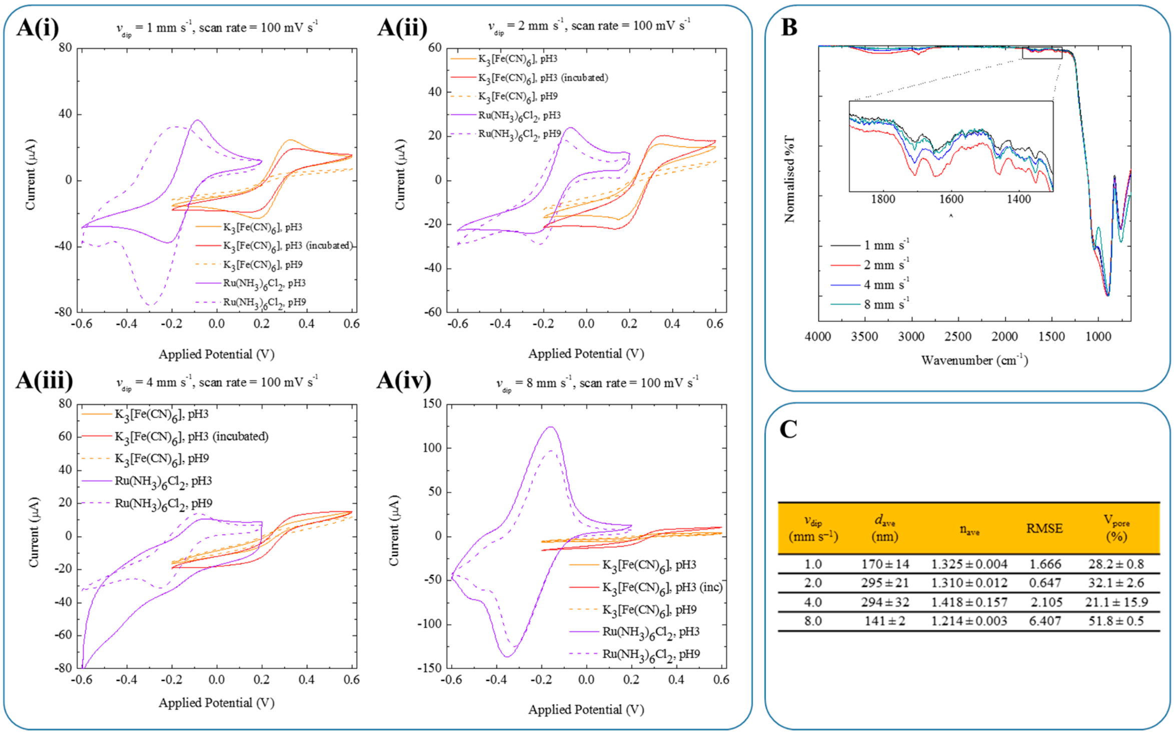

v_{dip} (mm s^{-1})	d_{ave} (nm)	n_{ave}	RMSE	V_{pore} (%)
1.0	170 ± 14	1.325 ± 0.004	1.666	28.2 ± 0.8
2.0	295 ± 21	1.310 ± 0.012	0.647	32.1 ± 2.6
4.0	294 ± 32	1.418 ± 0.157	2.105	21.1 ± 15.9
8.0	141 ± 2	1.214 ± 0.003	6.407	51.8 ± 0.5

Figure S8. Impact of withdrawal speed on the preparation of 5SBDCSi mesoporous films investigated by (**A**) CV (100 mVs^{-1}), (**B**) IR, and (**C**) ellipsometry. The dip-coating solution contained a TEOS:SDBC:F127:THF:H_2O:HCl molar ratio of 0.95:0.05:0.0075:20:5.2:0.28, and the films were prepared at 25 °C and 50% r.H. before being aged (24 h) and calcined at 60 °C (24 h), 130 °C (24 h), and finally at 200 °C (1 h).

To improve the dip-coating conditions further, the temperature (Figure S9) and relative humidity (data not shown) was also investigated.

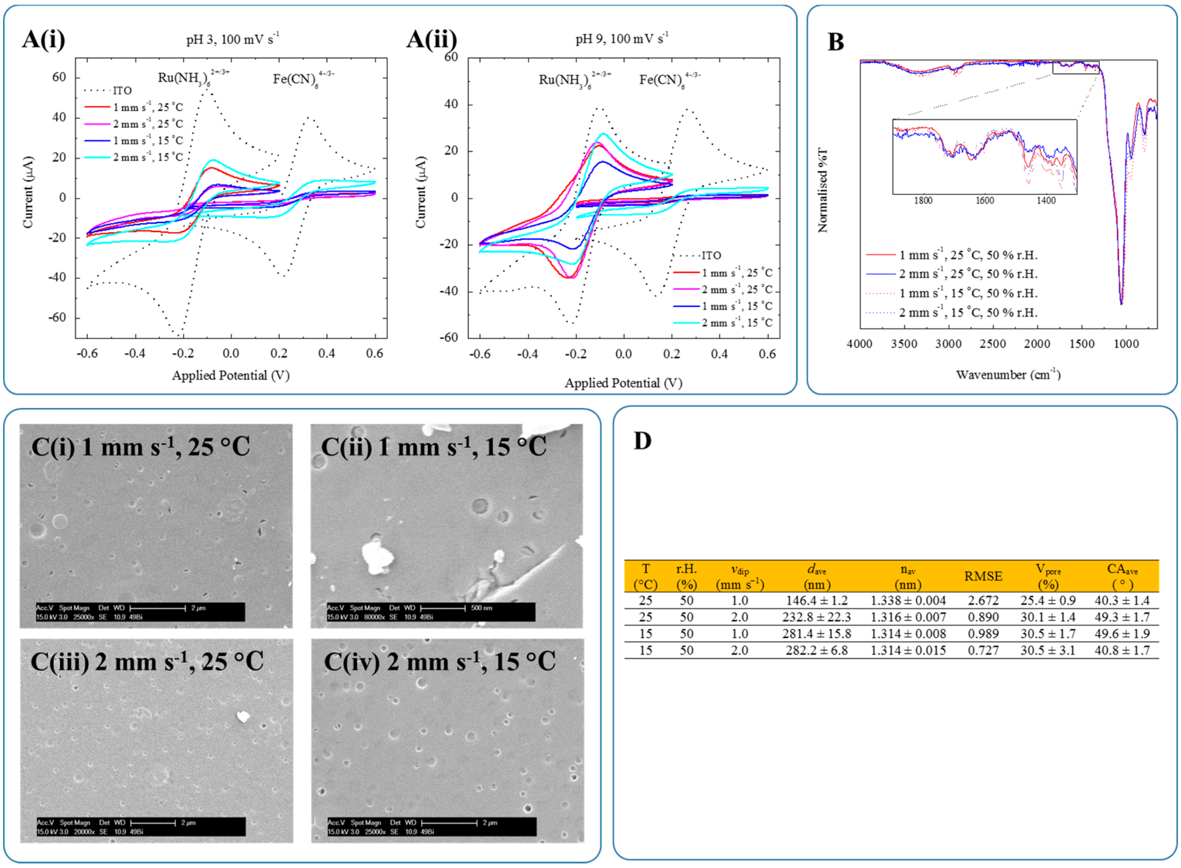

T (°C)	r.H. (%)	v_{dip} (mm s^{-1})	d_{ave} (nm)	n_{av} (nm)	RMSE	V_{pore} (%)	CA_{ave} (°)
25	50	1.0	146.4 ± 1.2	1.338 ± 0.004	2.672	25.4 ± 0.9	40.3 ± 1.4
25	50	2.0	232.8 ± 22.3	1.316 ± 0.007	0.890	30.1 ± 1.4	49.3 ± 1.7
15	50	1.0	281.4 ± 15.8	1.314 ± 0.008	0.989	30.5 ± 1.7	49.6 ± 1.9
15	50	2.0	282.2 ± 6.8	1.314 ± 0.015	0.727	30.5 ± 3.1	40.8 ± 1.7

Figure S9. Impact of withdrawal speed and temperature on the preparation of 5SBDCSi mesoporous films investigated by (**A**) CV (100 mV s^{-1}), (**B**) IR, (**C**) SEM, and (**D**) ellipsometry and static CA. The dip-coating solution contained a TEOS:SDBC:F127:THF:H_2O:HCl molar ratio of 0.95:0.05:0.0075:20:5.2:0.28, and the films were prepared at 50% r.H. before being aged (24 h) and calcined at 60 °C (24 h), 130 °C (24 h), and finally at 200 °C (1 h).

Based on the porosity, degree of functionality, film quality and ionic permselectivity, the preparation of 5SBDCSi films was carried out using a TEOS:SDBC:F127:THF:H_2O:HCl molar ratio of 0.95:0.05:0.0075:20:5.2:0.28 at 15 °C and a relative humidity of 50%. A summary of the film properties is given in Figure S10. The films display good ionic permselectivity towards both positive and negative charges, displaying behaviour typical of mesoporous silica with changes in pH (Figure S10 (a)). This behaviour can be attributed to the silica surface charge, moderate porosity of 21% (Figure S10 (b)), and the interconnected porous structure of the 5SBDCSi films with pores less than 10 nm in diameter as observed by TEM (Figure S10 (c)). The reduction in the contact angle from 45 to 27° upon exposure to a CO_2 plasma treatment indicates that the SBDC initiating sites are in fact accessible on the surface, and hence should be available and capable of undergoing polymerisation for localised SI-PIMP.

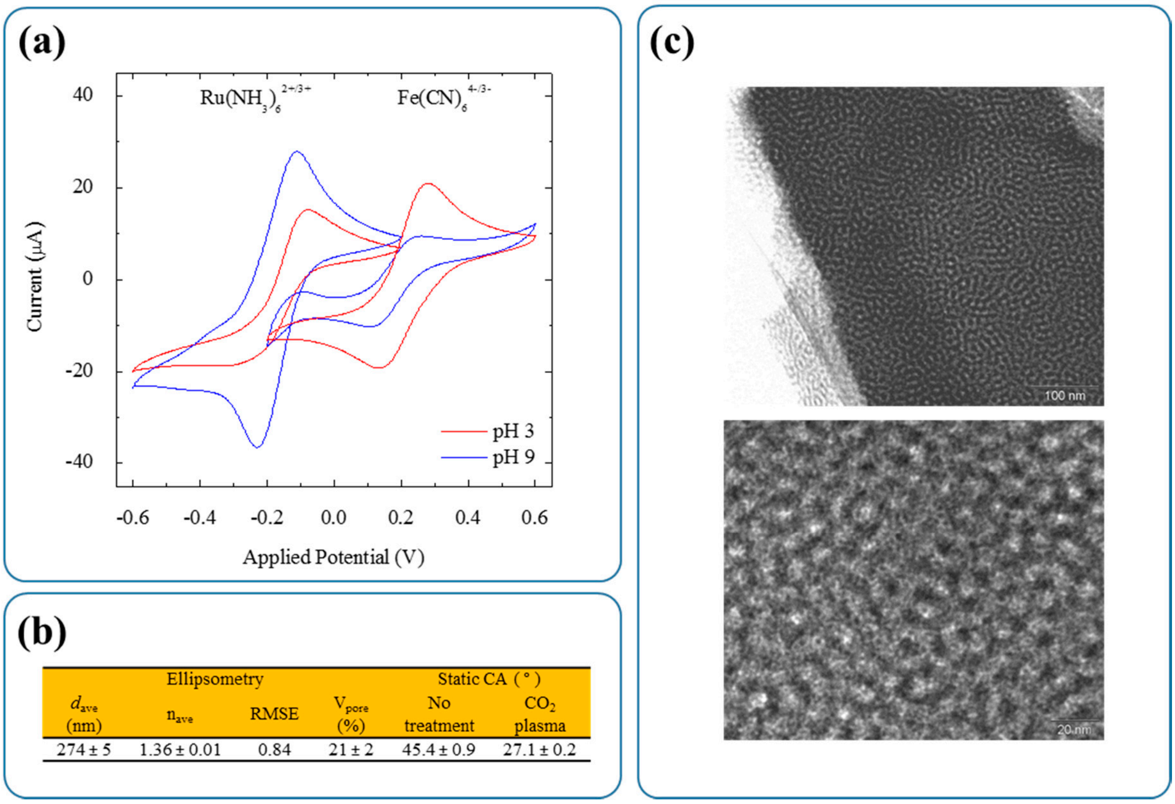

Figure S10. Mesoporous silica films prepared with 5 mol% SBDC, 5SBDCSi, at a temperature of 15 °C, a relative humidity of 50%, and a withdrawal speed of 2 mm s^{-1} display (**a**) good ionic pore selectivity towards both positive and negative charges, (**b**) a relativity high porosity, and (**c**) an interconnected mesoporous structure according to TEM observation. The CV traces shown in (a) were recorded at a scan rate of 100 mV s^{-1} in 100 mM KCl with either $Fe(CN)_6^{4-/3-}$ or $Ru(NH_3)_6^{2+/3+}$ at a concentration of 2 mM.

The co-condensation of SBDC resulted in a reduced specific surface area of approximately 4.9 m^2 g^{-1} compared to typical mesoporous silica films prepared using F127 as a templating agent (**Table S1**).

Table S1. Structural characterisation of mesoporous silica and 5SBDCSi by ellipsometry, TEM, and BET.

Material	d_{ave} (nm)	n_{ave} (nm)	RMSE	V_{pore} (%)	CA (°)	Pore size[1] (nm)	Neck Size[1] (nm)	Film[2] (s/g)	Film[2] (m^2/g)	Powder[2] (s/g)
*m*Si	180 ± 8	-	-	44	-	8	3-5	0.173	160.3	80.1
5SBDCSi	232 ± 3	1.36 ± 0.01	0.481	21 ± 2	50 ± 1	8	3-5	0.011	4.9	12.9

[1] Pore and neck size were approximated from TEM observation. [2] The specific surface area was determined by BET measurements at 77 K from the film on silicon wafer substrates and from powdered samples.

3b. Polymerisation Behaviour under Confinement and Membrane Permselectivity

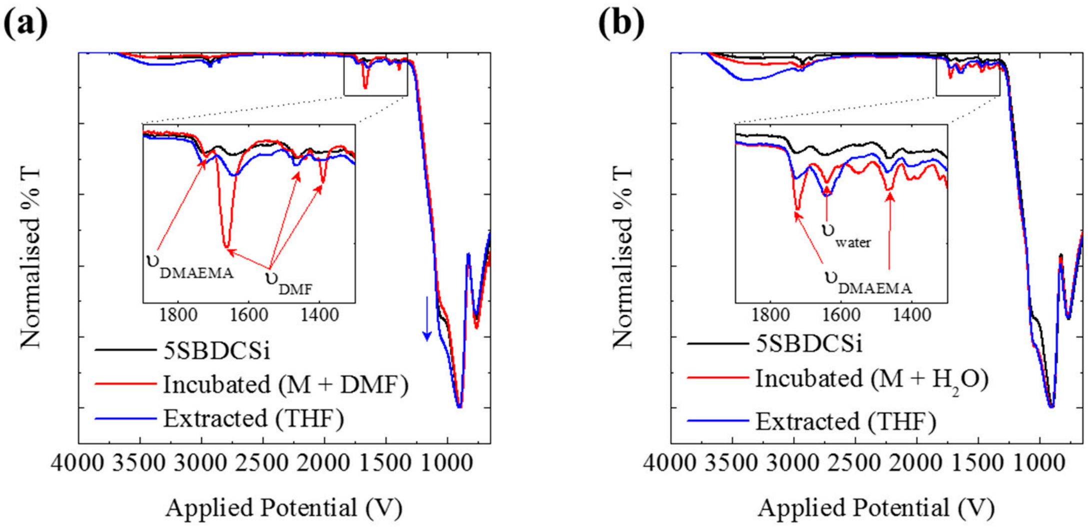

Figure S11. IR spectra showing that the diffusion of monomer, DMAEMA, is not hindered by wetting or electrostatics: the 5SBDCSi films were incubated in a mixture of DMAEMA in either (**a**) DMF or (**b**) H_2O for 1 h before being dried with pressurised air and extracted 3 times in THF.

Table S2. Average static contact angle of a 5SBDCSi film after performing a SIP for 0, 60, and 240 min exclusively with and without a CO_2 plasma treatment performed in the absence of a SI.

Polymerisation Time (min)	Static Contact Angle (°)	
	Pores Only	Pores + Exterior Surface
0	12 ± 1	50 ± 1
60	71 ± 1	78 ± 2
240	86 ± 1	79 ± 4

Table S3. Average static contact angle of a 5SBDCSi film after performing a SIP for 0, 60, and 240 min exclusively with and without a CO_2 plasma treatment performed in the presence of a SI.

Polymerisation Time (min)	Static Contact Angle (°)	
	Pores Only	Pores + Exterior Surface
0	12 ± 1	50 ± 1
60	49 ± 3	52 ± 2
240	53 ± 1	65 ± 2

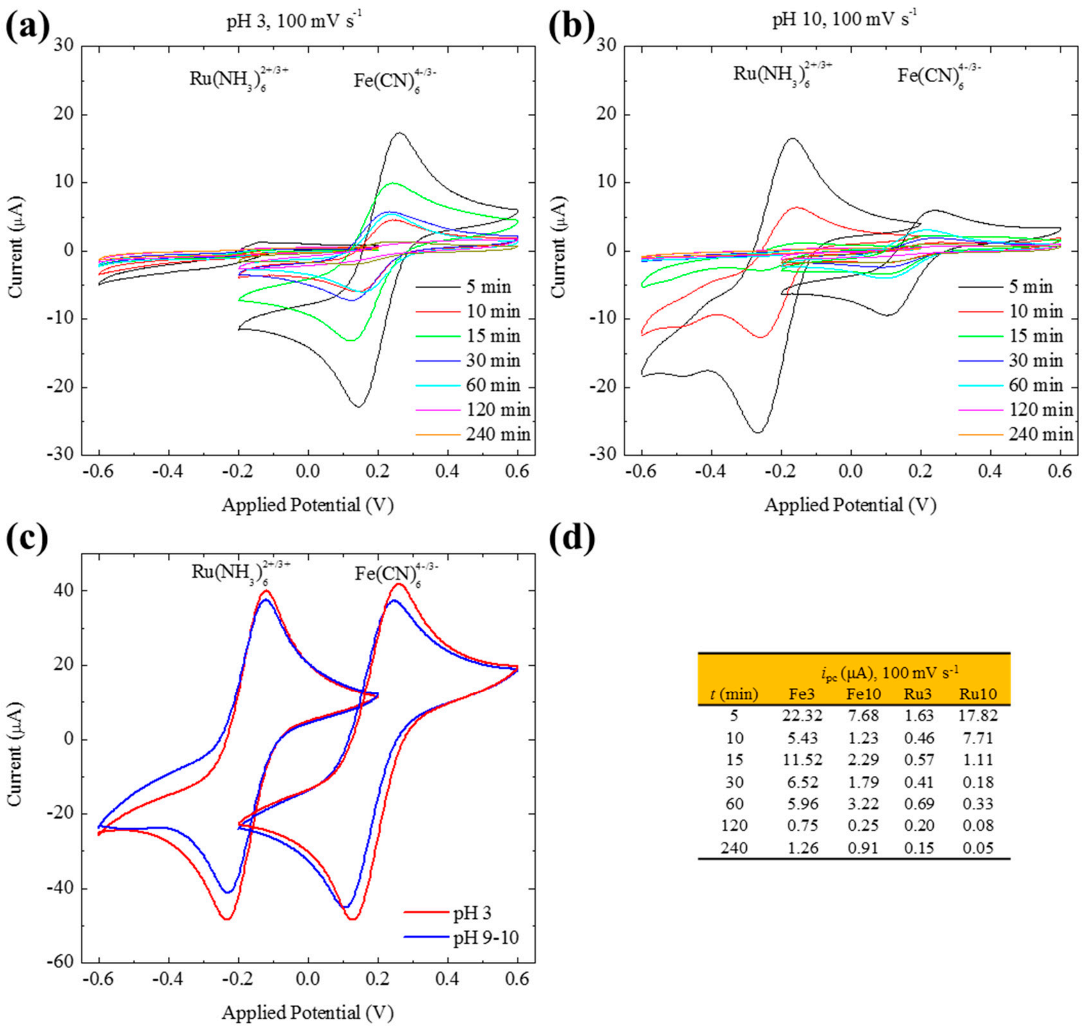

t (min)	i_{pc} (μA), 100 mV s^{-1} Fe3	Fe10	Ru3	Ru10
5	22.32	7.68	1.63	17.82
10	5.43	1.23	0.46	7.71
15	11.52	2.29	0.57	1.11
30	6.52	1.79	0.41	0.18
60	5.96	3.22	0.69	0.33
120	0.75	0.25	0.20	0.08
240	1.26	0.91	0.15	0.05

Figure S12. Ionic permselectivity of CO_2 plasma-treated 5SBDCSi films functionalised with PDMAEMA for varying polymerisation times at (**a**) pH 3 and (**b**) pH 10 in comparison to (**c**) bare ITO, and the corresponding (**d**) cathodic peak currents.

Table S4. Structural characterisation of 5SBDCSi with increasing SIP time with DMAEMA by ellipsometry and contact angle measurements. Reaction conditions: DMAEMA:DMF = 1:8 (v/v), 0–240 min, 320–400 nm.

t (min)	d_{ave} (nm)	n_{ave}	RMSE	V_{pore} (%)	Pore Filling (%)	CA (°)
0	274.0 ± 5.4	1.358 ± 0.012	0.840	21.3 ± 2.4	0	27 ± 0
5	280.8 ± 13.0	1.367 ± 0.010	0.594	19.6 ± 8.8	9.0 ± 6.4	56 ± 2
10	280.7 ± 9.5	1.390 ± 0.003	0.43	15.5 ± 0.5	28.3 ± 2.3	55 ± 3
15	259.2 ± 3.6	1.403 ± 0.024	0.595	13.0 ± 4.5	39.6 ± 20.7	56 ± 2
30	261.7 ± 7.3	1.450 ± 0.039	0.442	9.2 ± 6.6	71.2 ± 23.3	58 ± 3
60	296.2 ± 6.2	1.436 ± 0.022	0.475	6.8 ± 4.1	68.2 ± 19.2	63 ± 4
120	296.7 ± 2.0	1.460 ± 0.020	0.430	2.6 ± 3.0	87.8 ± 14.0	69 ± 7
240	320.2 ± 12.9	1.452 ± 0.020	0.716	3.9 ± 3.7	82.0 ± 17.3	79 ± 1

3c. 5SBDCSi Film Stability in Polymerisation Medium

CV measurements were conducted before and after incubation of 5SBDCSi in a mixture of DMAEMA and DMF. This was conducted to test whether the observed reduced permselectivity after polymerisation was the result of pore blocking due to polymer formation, or due to the stability of the film in the polymerisation mixture. Similar behaviour is observed before and after the incubation in the polymerisation mixture, indicating that the films remain stable over the period the polymerisation is conducted.

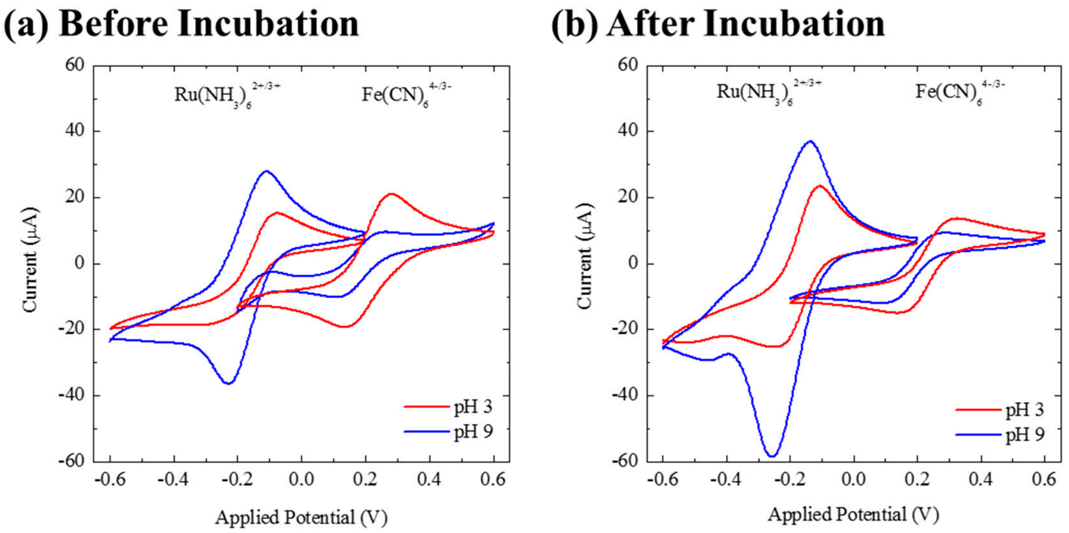

Figure S13. 5SBDCSi films were incubated in a 1/8 (v/v) mixture of DMAEMA in DMF to determine whether the film was stable under these conditions, and negate film degradation as the source of pore blocking. The samples were incubated in 100 mM KCl for 2 h before the CV measurements were conducted.

4. Re-Initiation

The mesoporous silica films for re-initiation experiments were prepared via sol–gel chemistry using tetraethoxysilane (TEOS) and Pluronic F127 in different ratios.

A typical synthesis of films with 8 nm (or 16 nm [48]) pores used 1.37 g (2.61 g) of Pluronic F127, which was dissolved in 33.8 mL (24.0 mL) of absolute ethanol and 5.22 mL of H_2O, 0.33 mL of 37% HCl (6.4 mL of 0.05 M HCl). After which, 6.55 mL (4.88 mL) of TEOS was added to the mixture, and the solution was then stirred overnight under ambient conditions.

Dip-coating was performed in a climate-controlled chamber at a temperature of 25 °C and a relative humidity of 50% at a withdrawal speed of 2 mm s^{-1}. After aging the films for 1 h they were subjected to the following thermal treatment: Two 1 h steps at 60 and 130 °C followed by heating to 350 °C at a heating rate of 1 °C/min. Finally, the films were stabilised at 350 °C for 2 h.

The mesoporous silica films were then post-grafted with SBDC under an inert atmosphere. SBDC (7.2 mg, 0.02 mmol) was dissolved in absolute toluene (20.0 mL), and the mixture heated to 80 °C for 60 min in the presence of the mesoporous films. The films were then extracted through multiple washings with toluene.

The first surface-initiated polymerisation, SIP (1), was performed as described in the Experimental Section (Section 2.3.4). For the subsequent SIP, SIP (2), MEP (0.55 g) was dissolved in H_2O (20 mL). The substrate was then added to this solution before the mixture was sealed using a rubber septum and deoxygenated for 15 min before irradiation (t = 15 min, λ = 365 nm, P = 40 W). The substrates were then extracted in a water bath overnight.

Table S5. Investigating the pore filling from 8 nm pore mesoporous silica post-grafted with SBDC by ellipsometry. The position indicates the measurement position on the film (i) before modification, and after polymerisation with (ii) DMAEMA and re-initiation with (iii) MEP. The polymerisation was confined to the pores by performing a CO_2 plasma treatment prior to polymerisation. Reaction conditions for SIP (1): DMAEMA:DMF = 1:8 (v/v), t = 5 min, λ = 365 nm, P = 40 W. Reaction conditions for SIP (2): [MEP] = 0.125 M, solvent = water, t = 15 min, λ = 365 nm, P = 40 W. The free volume was estimated assuming the refractive index of the polymer was 1.517 (PDMAEMA).

Position	Step	***d*** (nm)	***n***	RMSE	Free Volume (%)
	i	155 ± 0.1	1.275 ± 0.000	0.172	39
1	ii	155 ± 0.2	1.376 ± 0.001	0.543	20
	iii	157 ± 0.5	1.453 ± 0.002	1.426	5.5
	i	160 ± 0.2	1.274 ± 0.001	0.347	39
2	ii	162 ± 0.4	1.370 ± 0.002	1.059	21
	iii	159 ± 0.5	1.445 ± 0.000	0.264	7
	i	163 ± 0.1	1.265 ± 0.000	0.115	41
3	ii	163 ± 0.1	1.376 ± 0.000	0.231	20
	iii	168 ± 0.8	1.445 ± 0.004	1.464	7

Table S6. Investigating the pore filling from 16 nm pore mesoporous silica post-grafted with SBDC by ellipsometry. The position indicates the measurement position on the film (i) before modification, and after polymerisation with (ii) DMAEMA and re-initiation with (iii) MEP. The polymerisation was confined to the pores by performing a CO_2 plasma treatment prior to polymerisation. Reaction conditions for SIP (1): DMAEMA:DMF = 1:8 (v/v), t = 5 min, λ = 365 nm, P = 40 W. Reaction conditions for SIP (2): [MEP] = 0.125 M, solvent = water, t = 15 min, λ = 365 nm, P = 40 W. The free volume was estimated assuming the refractive index of the polymer was 1.517 (PDMAEMA).

Position	Step	*d* (nm)	*n*	RMSE	Free Volume (%)
	i	592 ± 5	1.186 ± 0.005	2.651	58
1	ii	536 ± 5	1.408 ± 0.008	1.081	16
	iii	563 ± 2	1.452 ± 0.004	0.508	7.7
	i	589 ± 9	1.208 ± 0.008	4.790	53
2	ii	520 ± 4	1.410 ± 0.006	1.173	15
	iii	559 ± 2	1.459 ± 0.003	0.390	5.9
	i	589 ± 5	1.177 ± 0.004	2.375	60
3	ii	512 ± 2	1.402 ± 0.004	1.018	17
	iii	555 ± 2	1.445 ± 0.003	0.373	8.0

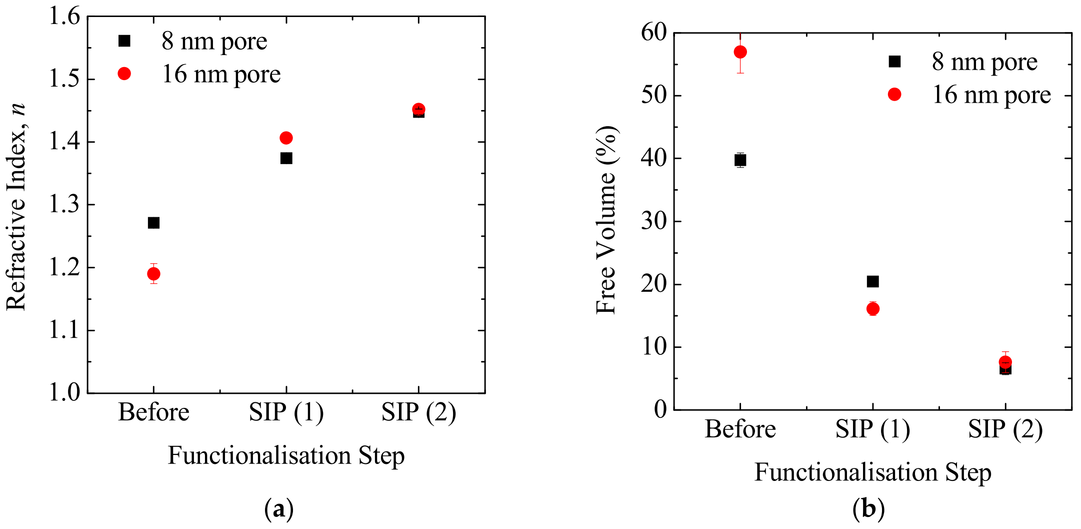

Figure S14. (**a**) Refractive index and corresponding (**b**) free volume before and after performing a SIP with DMAEMA and chain-extension with MEP. The mesoporous silica films were characterised by 8 and 16 nm pores, and were post-grafted with SBDC. Reaction conditions for SIP (1): DMAEMA:DMF = 1:8 (v/v), t = 5 min, λ = 365 nm. Reaction conditions for SIP (2): [MEP] = 0.125 M, solvent = water, t = 15 min, λ = 365 nm. The free volume was estimated assuming the refractive index of the polymer was 1.517 (PDMAEMA).

5. Calculation of the Porosity and Pore Filling using the Brüggemann Effective Medium Approximation [32,33]

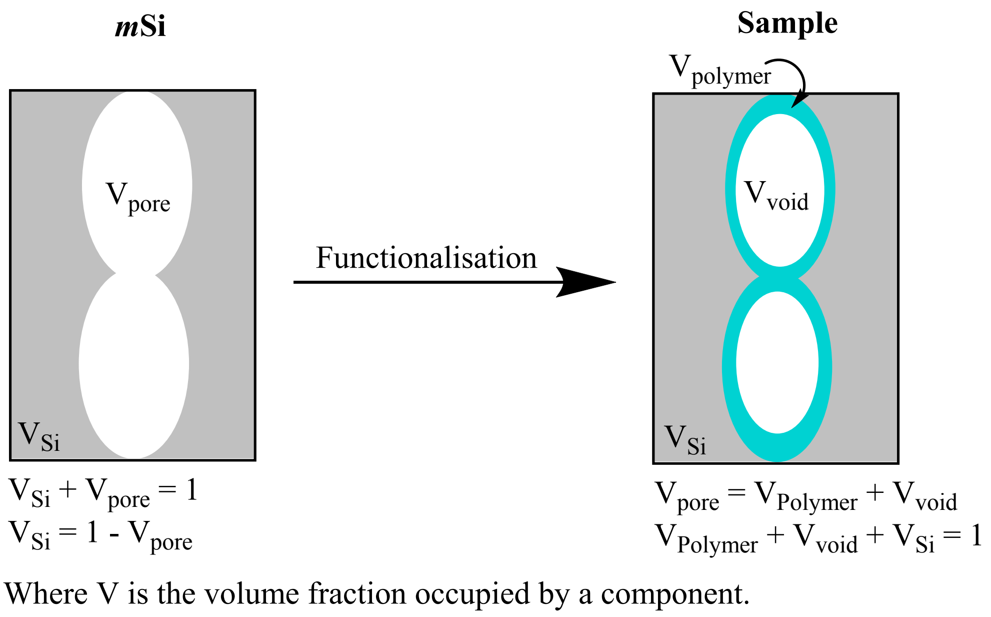

Scheme S1. Diagram depicting the mesoporous silica film before and after polymerisation: V_{Si} and V_{pore} correspond to the volume fraction occupied by silica and the layer porosity of empty silica before functionalisation; $V_{polylmer}$ and V_{void} correspond to the volume fraction occupied by polymer and free volume remaining after functionalisation.

5a. Calculating the Material Porosity of Empty Mesoporous Silica (mSi):

According to the Brüggemann Effective Medium Approximation, the following relationship exists between the dielectric constants and volume fraction of each constituent material:

$$V_{pore}\left(\frac{\varepsilon_{pore}-\varepsilon_{mSi}}{\varepsilon_{pore}+2\varepsilon_{mSi}}\right)+V_{Si}\left(\frac{\varepsilon_{Si}-\varepsilon_{mSi}}{\varepsilon_{Si}+2\varepsilon_{mSi}}\right)=0 \quad \text{(S1)}$$

where V_{pore} and V_{Si} correspond to the volume fractions occupied by air and silica, and ε_{pore}, ε_{Si}, and ε_{mSi} correspond to the dielectric constants of air, silica, and the mesoporous silica sample (n calculated from ellipsometry).

Since V_{Si} = 1 – V_{pore}, and the dielectric constant of both air (n = 1) and silica (n = 1.46) can be calculated from their known refractive indices ($\varepsilon = n^2$), we can write (S1) as follows:

$$V_{pore}\left(\frac{1-n_{mSi}^2}{1+2n_{mSi}^2}\right)+(1-V_{pore})\left(\frac{2.1316-n_{mSi}^2}{2.1316+2n_{mSi}^2}\right)=0 \quad \text{(S2)}$$

By rearranging Equation (S2), we can obtain the following expression to calculate the porosity using the refractive index calculated from ellipsometry of the mesoporous silica thin film:

$$V_{pore}=\frac{y}{y-x} \quad \text{(S3)}$$

where $x=\left(\frac{1-n_{mSi}^2}{1+2n_{mSi}^2}\right)$, and $y=\left(\frac{2.1316-n_{mSi}^2}{2.1316+2n_{mSi}^2}\right)$

5b. Calculating the Pore Filling of Mesoporous Silica with Polymer

After functionalisation with polymer, the following expression can be written in accordance with the Brüggemann Effective Medium Approximation:

$$V_{void}\left(\frac{\varepsilon_{void}-\varepsilon_{sample}}{\varepsilon_{void}+2\varepsilon_{sample}}\right)+V_{polymer}\left(\frac{\varepsilon_{polymer}-\varepsilon_{sample}}{\varepsilon_{polymer}+2\varepsilon_{sample}}\right)+V_{Si}\left(\frac{\varepsilon_{Si}-\varepsilon_{sample}}{\varepsilon_{Si}+2\varepsilon_{sample}}\right)=0 \qquad \text{(S4)}$$

The volume fraction of polymer, $V_{polymer}$, can be determined by substituting $V_{void} = V_{pore} - V_{polymer}$ into Equation (S4), followed by rearrangement to give the following expression:

$$V_{polymer}=\frac{aV_{pore}+cV_{Si}}{(a-b)} \qquad \text{(S5)}$$

where $a=\left(\frac{1-n_{sample}^2}{1+2n_{sample}^2}\right)$, $b=\left(\frac{2.301-n_{sample}^2}{2.301+2n_{sample}^2}\right)$, and $c=\left(\frac{2.132-n_{sample}^2}{2.132+2n_{sample}^2}\right)$ for PDMAEMA (n = 1.517).

The volume occupied by the polymer in the mesopores can then be calculated as follows:

$$\text{Pore Filling}=\frac{\text{V}_{\text{polymer}}}{\text{V}_{\text{pore}}}\times 100 \qquad \text{(S6)}$$

Where $V_{polymer}$ corresponds to the volume fraction occupied by the polymer in the sample ($V_{polymer} + V_{void} + V_{Si} = 1$), and V_{pore} is the porosity of the empty mesoporous silica prior to functionalisation.
